# Chromosomal instability and a deregulated cell cycle are intrinsic features of high‐risk gastrointestinal stromal tumours with a metastatic potential

**DOI:** 10.1002/1878-0261.13514

**Published:** 2023-09-03

**Authors:** Heidi Maria Namløs, Ksenia Khelik, Sigve Nakken, Daniel Vodák, Eivind Hovig, Ola Myklebost, Kjetil Boye, Leonardo A. Meza‐Zepeda

**Affiliations:** ^1^ Department of Tumor Biology, Institute for Cancer Research, The Norwegian Radium Hospital Oslo University Hospital Oslo Norway; ^2^ Centre for Cancer Cell Reprogramming, Faculty of Medicine, Institute of Clinical Medicine University of Oslo Oslo Norway; ^3^ Department of Informatics University of Oslo Oslo Norway; ^4^ Bioinformatics Core Facility, Department of Core Facilities, Institute for Cancer Research Oslo University Hospital Oslo Norway; ^5^ Department for Clinical Science University of Bergen Bergen Norway; ^6^ Department of Oncology Oslo University Hospital Oslo Norway; ^7^ Genomics Core Facility, Department of Core Facilities, Institute for Cancer Research Oslo University Hospital Oslo Norway

**Keywords:** chromothripsis, CIN, GIST, LOH, miR‐34a, risk classification

## Abstract

Patients with localised, high‐risk gastrointestinal stromal tumours (GIST) benefit from adjuvant imatinib treatment. Still, approximately 40% of patients relapse within 3 years after adjuvant therapy and the clinical and histopathological features currently used for risk classification cannot precisely predict poor outcomes after standard treatment. This study aimed to identify genomic and transcriptomic profiles that could be associated with disease relapse and thus a more aggressive phenotype. Using a multi‐omics approach, we analysed a cohort of primary tumours from patients with untreated, resectable high‐risk GISTs. We compared patients who developed metastatic disease within 3 years after finishing adjuvant imatinib treatment and patients without disease relapse after more than 5 years of follow‐up. Combining genomics and transcriptomics data, we identified somatic mutations and deregulated mRNA and miRNA genes intrinsic to each group. Our study shows that increased chromosomal instability (CIN), including chromothripsis and deregulated kinetochore and cell cycle signalling, separates high‐risk samples according to metastatic potential. The increased CIN seems to be an intrinsic feature for tumours that metastasise and should be further validated as a novel prognostic biomarker for high‐risk GIST.

## Graphical abstract

Through multi‐omics analysis of localised, high‐risk gastrointestinal stromal tumours, we uncovered intrinsic properties associated with metastatic potential. Patients who developed metastatic disease within 3 years after adjuvant imatinib treatment had a higher level of chromosomal instability and deregulated cell cycle processes compared to patients without disease relapse for more than 5 years after the end of treatment.

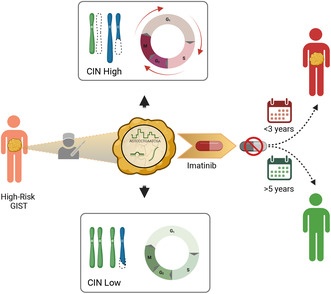

**Abbreviations**
CIN: chromosomal instability
CNV: copy number variation
GIST: Gastrointestinal Stromal Tumour
IPA: Ingenuity Pathway Analysis
logFC: log fold change
LOH: loss of heterozygosity
MAF: mutant allele frequency
Met: metastatic
NoMet: non‐metastatic
SNV: single nucleotide variants
TPM: transcripts per million

## 1 Introduction

Gastrointestinal stromal tumour (GIST) is the most frequent soft tissue sarcoma and is characterised by activating mutations in the receptor tyrosine kinases *KIT* or *PDGFRA*. GIST represents a paradigm for the development of precision cancer medicine, where a successful long‐time response to targeted treatment by tyrosine kinase inhibitors is achieved for many patients. Adjuvant treatment with imatinib for three years is recommended for patients with localised GIST who have a significant risk of relapse [1]. The prognosis and treatment decisions after curatively intended resection rely on the classification of recurrence risk according to defined criteria including tumour size, mitotic index, tumour rupture and anatomical site [2, 3]. Several risk classifications have been proposed, of which the most commonly used are the modified NIH (mNIH) classification and the Armed Forces Institute of Pathology (AFIP) criteria [2]. Patients classified as high‐risk benefit from adjuvant imatinib treatment, but around 40% of the patients relapse within three years after stopping the recommended three years of treatment [4]. Furthermore, historical data have shown that almost half of the high‐risk patients could be cured by surgery alone [5]. Thus, the high‐risk group includes both tumours with a good prognosis without imatinib and tumours that recur despite adjuvant treatment, and there is a need for an improved understanding of the biological differences.

The gain of oncogenic mutations in *KIT* or *PDGFRA* is the initial event in GIST [6, 7]. Most GISTs then progress through a stepwise accumulation of large‐scale genomic gains, losses and rearrangements, known as chromosomal instability (CIN). Studies have shown that the number of genomic changes can predict the clinical outcome of intermediate‐risk patients as defined by AFIP [8, 9]. We have recently shown for the first time that genomic complexity is a prognostic biomarker in high‐risk GIST and that tumour recurrences are infrequent for patients with a simple tumour karyotype [10]. Models introducing CIN as a prognostic biomarker may provide a more accurate estimation of the risk of metastasis, but a more detailed understanding of the molecular differences between genomically simple and complex tumours is needed.

The accumulation of chromosomal aberrations is associated with the inactivation of cell cycle control genes such as *TP53*, *RB1* and *CDKN2A* [11]. Mutation of the KIT and PDGF receptors leads to a constitutional activation and ligand‐independent downstream signalling through the RAS/RAF/MAPK, PI3K/AKT/mTOR and STAT3 pathways [12, 13]. These pathways further upregulate important transcriptional activators, stimulating the cell cycle and having anti‐apoptotic effects [14, 15, 16, 17, 18, 19, 20, 21]. The cell cycle pathways are part of complex interacting networks with miRNAs as central regulatory functions [22]. The integration of the various sources of genomic data may help in understanding the intrinsic biology of GIST. Thus, the observed biological and clinical behaviour differences can be better understood.

This study aimed to characterise the genomic and transcriptomic landscape of high‐risk GIST to increase the understanding of the differences between tumours that metastasize and those that do not recur. We investigated somatic mutations, DNA copy number changes, and expression of mRNA and miRNA genes and pathways. This showed that CIN and deregulation of cell cycle pathways were the most prominent features associated with increased metastatic capacity in high‐risk GIST.

## 2 Materials and methods

### 2.1 Tumour patient characteristics

The “GIST Risk” patient cohort consisted of 21 patients with resectable, gastric, high‐risk GIST, where primary, untreated tumours were collected. Clinicopathological characteristics are summarised in Table 1. None of the patients received neoadjuvant therapy before tumour resection. Sixteen patients received adjuvant imatinib treatment for a median duration of 32 months (range 2–60 months). Disease recurrence was recorded for 10 patients. All developed metastases to the liver and/or peritoneum without locoregional recurrences. The median time from primary tumour surgery to metastasis was 27 months (range 6–63 months). Median follow‐up for patients without metastasis was 112 months (range 85–134 months).

**Table 1:** Clinical and histopathological characteristics based on whether patients developed metastases during follow‐up. Risk classification was performed at the time of primary tumour surgery or diagnosis. HPF, high‐power field of the microscope.

	Number of patients (%)	
	Non‐metastatic	Metastatic
Age (years)^a^	71 (38–81)	67 (39–82)
Sex		
Female	7 (64)	2 (20)
Male	4 (36)	8 (80)
Tumour location		
Stomach	11 (100)	10 (100)
Tumour size (cm)^a^	9.0 (6.0–20.0)	12.5 (6.5–28.0)
Mitoses per 50 HPF^a^	7 (1–130)	28 (3–178)
Tumour rupture		
Yes	2 (18)	7 (70)
No	9 (82)	3 (30)
Adjuvant imatinib treatment	10 (91)	6 (60)
Mutation analysis		
*KIT* exon 11 del557/558	5 (45)	8 (80)
*KIT* exon 11 other	4 (36)	1 (10)
*PDGFRA* exon 18	2 (18)	1 (10)

^a^ Values are median (range).

The patient cohort was divided into two groups: (i) 10 high‐risk GIST patients who developed metastatic disease within three years after the end of imatinib treatment or surgery for patients without adjuvant imatinib, and (ii) 11 high‐risk GIST patients without disease relapse after at least five years of follow‐up after the end of imatinib treatment or surgery for patients without adjuvant imatinib. Fresh frozen tumour tissues and blood samples for germline control were available for all patients. The tumours were diagnosed using the current World Health Organization classification and risk classified using the modified mNIH criteria [2]. The samples were collected between 2002 and 2017 at Oslo University Hospital (OUH). Written informed consent was obtained from all patients enrolled in the study. The study was approved by the Regional Committees for Medical Research Ethics Southern and Eastern Norway (Project S‐06132). The study was performed according to the Declaration of Helsinki.

### 2.2 Isolation of nuclei acids

Genomic DNA and total RNA, including miRNAs, were extracted from fresh frozen tumour samples using the Qiagen AllPrep DNA/RNA/miRNA Universal kit according to the manufacturer's instructions (Qiagen, GmBH, Hilden, Germany). As a germline control, peripheral blood mononuclear cells' genomic DNA was isolated using the Qiagen QIAmp DNA blood kit.

### 2.3 Whole exome sequencing

The whole genome sequencing libraries were generated using the Agilent SureSelectXT reagent kit according to the manufacturer's instructions. Furthermore, exome sequences were captured using the Agilent Human All Exon V5 capturing probe set (Agilent, Santa Clara, CA, USA). Exome libraries were sequenced paired‐end 2x150 bp on the Illumina HighSeq 4000 instrument. Library preparation and sequencing were performed by the Oslo University Hospital [Genomics Core Facility (Oslo.genomics.no)]. The mean coverage of the target regions for tumour and peripheral white blood cell control samples were 449× and 196×, respectively.

### 2.4 Somatic and germline variant detection

A benchmarked in‐house bioinformatics pipeline [23] was used to process the sequencing reads and identify somatic changes. Sequence alignment was performed using BWA MEM [24] towards the b37 genome assembly with added decoy contigs. Further pre‐processing was performed with picard (http://broadinstitute.github.io/picard/) and GATK [25] before performing the calling of somatic single nucleotide variants (SNVs) and insertions/deletions (INDELs) using MuTect [26] and Strelka [27]. Variant calling files were further processed and annotated using PCGR [28]. The somatic variants were further filtered according to the following criteria: tumour sample coverage ≥ 50×, control sample coverage ≥ 30×, tumour sample mutant allele frequency (MAF) ≥ 0.03, and number of reads supporting MAF in the tumour sample ≥ 5. Protein‐coding variants, including non‐synonymous, frameshift, and splice‐site mutations, were classified according to a four‐tiered structure of clinical significance [29]. Mutated genes were annotated as tumour suppressors and/or proto‐oncogenes using the Network of Cancer Genes (NCG) database [30]. The long KIT duplications were manually identified using IGV [31]. The Mann–Whitney test was used to determine significant differences in the number of SNVs. Tumour mutational burden (TMB) was estimated using PCGR, following the approach outlined in [32]. Low TMB was defined as fewer than 5 mutations per Mb [32].

Germline variants (SNVs/INDELs) were identified from the blood samples through the Illumina DRAGEN germline pipeline v.3.9 (07.021.595.3.7.5). Variant calling files were further processed and annotated using the Cancer Predisposition Sequencing Reporter (CPSR v0.6.1) [33] to identify potential cancer‐predisposition variants. CPSR was configured to report variants of clinical significance in an exploratory panel of 335 cancer predisposition genes.

### 2.5 Copy number analysis and chromosomal instability

Allele‐specific DNA copy‐number variation and frequency analysis were performed using FACETS [34]. Identification of significant recurrent copy‐number changes at the arm and focal level was performed by gistic v2.0 [35], where regions of alteration with a *q*‐value less than 0.25 were reported as significant. Genes corresponding to the significantly recurrent changed regions were reported. CIN was scored using the CINmetrics package (https://github.com/lasseignelab/CINmetrics). The scoring was based on the number of aberrant chromosomes containing regions with gain and loss of alleles and/or containing regions with copy‐neutral loss of heterozygosity (LOH). Identification of chromothripsis was performed per sample by counting the number of switches between copy‐number states for each chromosome based on the FACETS results. Chromosomes containing 10 or more such switches within a 50 Mb interval were classified as chromothripsis‐positive with high confidence, as described in [36].

### 2.6 mRNA sequencing analysis

RNA sequencing libraries were constructed using the KAPA RNA Hyper kit (Roche, Bassel, Switzerland) to generate total RNA libraries and the Twist Core Exome probe set (Twist Biosciences, San Francisco, CA, USA) to capture assay targets. RNA‐exome libraries were sequenced paired‐end 2x75 bp on an Illumina HiSeq 4000 instrument. The library preparation and sequencing were performed at the Oslo University Hospital Genomics Core Facility.

Sequencing reads were aligned to the GRCh38 human genome assembly using STAR [37] and gene expression quantification was performed using the *featureCounts* function of the subread package [38]. Genes with a median number of transcripts per million (TPM) < 2 in both metastatic and non‐metastatic groups were considered as being expressed below the background level and were filtered out. Differentially gene expression (DE) analysis was performed using the DESeq2 r package [39]. Genes with an adjusted *P*‐value < 0.05 and absolute values of log_2_ fold change (logFC) > 1 were considered differentially expressed between the groups.

The differentially expressed genes between metastatic and non‐metastatic samples were analysed using QIAGEN Ingenuity Pathway Analysis (IPA) [40]. The analysis identified enriched biological processes (cut‐off |*z*| > 1.6 and Benjamini‐Hochberg adjusted *P*‐value < 0.05) and upstream regulators and biological functions (cut‐off |*z*| > 2 and Benjamini‐Hochberg adjusted *P*‐value < 0.05) among the sample groups.

A gene set enrichment analysis (GSEA) was performed using the fgsea r package [41]. The lfcShrink function from DeSeq2 was used for ranking. The GSEA was run using the cell proliferation gene set (cell_proliferation_GO_0008283) from the Human Molecular Signatures Database (MSigDB) [42].

### 2.7 Allele‐specific expression at mutated loci

Allele‐specific expression analysis of protein‐coding SNV was performed using the ASEReadCounter from GATK [25], which resulted in sample‐wise RNA read counts for mutated and wild‐type alleles for each input SNV. Before running the tool, the RNA sequencing reads were mapped to the GRCh37 human genome assembly with an added decoy using STAR and post‐processed using Picard. ASEReadCounter was run with the additional parameters “min‐mapping‐quality” of 10 and “min‐base‐quality” of 2. The expression counts for mutated and wild‐type alleles for protein‐coding INDELs were identified manually using IGV. The mutations were further classified into three categories: mutated allele expressed, mutated allele not expressed or mutated gene not expressed, based on TPM values of corresponding genes and variant allelic fractions (VAF) in the tumour. The SNVs not present in the covered RNA data were filtered out. A mutation was called expressed if the corresponding gene was expressed with a TPM ≥ 2 and VAF given by mRNA data was greater than 3%. If the corresponding gene was expressed, but VAF was ≤ 3%, then the mutated allele was classified as not expressed. If the corresponding TPM value was less than 2, the gene was considered as not expressed.

### 2.8 miRNA expression analysis

miRNA expression profiling was performed using the Human V3 miRNA assay from nanoString (nanoString Technologies Inc., Seattle, Washington, USA). The assay was performed by the Oslo University Hospital Genomics Core Facility. Quality control and preprocessing steps were performed using nSolver software from nanoString. Normalisation and differentially expressed gene analysis were performed using the NanostringDiff r package [43]. Genes with median read count expression values before and after normalisation less than 45 in both metastatic and non‐metastatic groups were filtered out. Genes with *q*‐value ≤ 0.05 and an absolute value of logFC ≥ 1 were considered differentially expressed.

miRNA target predictions were performed using the miRNA and mRNA interaction databases in IPA. miRNAs and putative mRNA targets were selected to have opposite expression fold changes, and the confidence level of the target predictions was set to experimentally observed and highly predicted interactions.

## 3 Results

### 3.1 The expressed mutational landscape of GIST

**Fig. 1:**
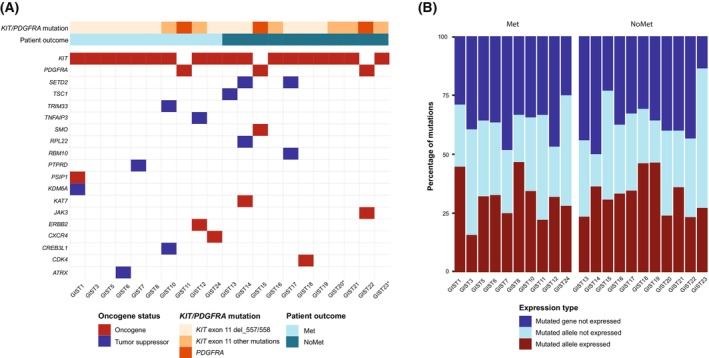
The expressed tumour mutational burden of GIST. (A) Topography of expressed mutations in tumour suppressor and oncogenes in GIST. (B) Sample‐wise proportion of the mutated loci with the expression of the mutated allele. Met: metastatic. NoMet: non‐metastatic.

By whole‐exome sequencing, a total of 738 somatic protein‐changing mutations (668 SNVs and 70 INDELs, Data S1) within the coding regions of 585 genes were found across the 21 tumours analysed. To explore the differences in behaviour and clinical outcome within the high‐risk group, we compared tumours from patients who developed metastasis to those without after long‐term follow‐up (> 5 years). Three tumours (14%) had oncogenic *PDGFRA* exon 18 mutations, and 18 tumours (86%) had *KIT* exon 11 mutations (Fig. 1A). Except for the *KIT* and *PDGFRA* mutations, no other mutations with clinical or potential clinical significance were observed among the samples. All tumours had a low tumour mutational burden (TMB < 3), but tumours from patients who developed a metastatic disease had a higher number of variants compared to non‐metastatic patients (39 vs 32 variants; *P* = 0.04). No pathogenic or likely pathogenic mutations associated with predisposition to GIST were observed.

Combined mutation and allele‐specific expression data were available for 719 mutated protein‐coding variants, where an average of 36% of the variants across the samples was found not to be expressed, and for 32%, only the wild‐type allele was expressed. Thus, only 230 of the somatic mutations (185 mutated SNVs and 45 INDELs) were found to be expressed within the GIST cohort. The average proportion of variants with expressed mutated alleles was 31% in metastatic and 33% in non‐metastatic samples (Fig. 1, Data S1). Among the total 585 genes, 68 were annotated as tumour suppressor genes and/or oncogenes. Of these, 19 genes were expressed; 10 tumour suppressors and 9 oncogenes were distributed evenly across the groups (Fig. 1).

### 3.2 High level of chromosomal instability is associated with the development of metastatic disease

**Fig. 2:**
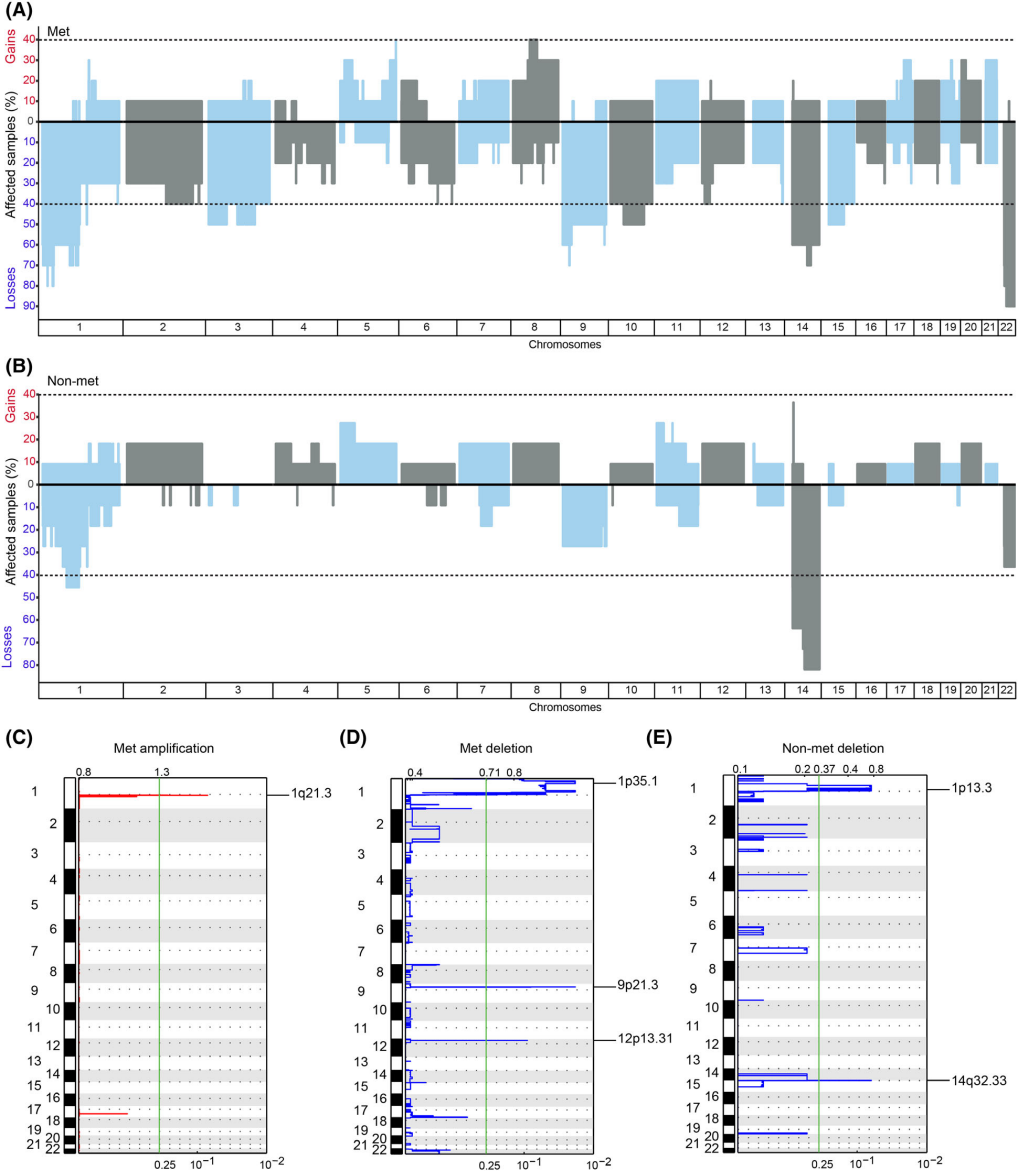
Identification of copy number changes in GIST. Frequency plots with an overview of deletions and amplifications for (A) metastatic samples (B) non‐metastatic samples. Blue and grey represent alternating chromosomes, and the genomic locations of chromosomal peaks are shown with the chromosome number along the y‐axis. Significant, recurrent arm‐level and focal genomic changes were seen across the GIST samples e.g. as shown in (C) amplification in metastatic samples (D) deletion in metastatic samples, and (E) deletion in non‐metastatic samples. The statistical significance of the aberrations is displayed as FDR *q* values along the *x*‐axis (cut‐off 0.25). The dotted lines show the centromere location. Met: metastatic. Non‐met: non‐metastatic.

The genomic copy number patterns were analysed using two different approaches, focusing on large‐scale or more segmental patterns. DNA copy number variation (CNV) analysis showed that ≥ 40% of metastatic samples had large‐scale amplification in chromosome 8 and deletions in chromosomes 1, 2, 3, 9, 10, 12, 14, 15 and 22 (Fig. 2A). Meanwhile, the non‐metastatic group only showed deletions in chromosomes 1 and 14 (Fig. 2B), providing different profiles for the two groups. Three of the metastatic and one of the non‐metastatic tumours did not have a deletion of chromosome 14. Recurrent focal and arm‐level copy number changes as determined by GISTIC were amplification of chromosome 1q and deletions in 1p, 9p and 12p in the metastatic group and deletions of 1p and 14q in the non‐metastatic group (Fig. 2C–E). The deleted regions contained the tumour suppressors *CDKN2C*, *SDHB*, and *SFPQ* in 1p and *CDKN2A* in 9p in metastatic samples and the tumour suppressors *BCL10* and *NOTCH2* and the oncogenes *JAK1* and *NRAS* in 1p in the non‐metastatic sample group (Data S2). The dystrophin (*DMD*) gene, previously reported to be frequently deleted in metastatic GISTs [44], was only found to be deleted in chromosome X in three samples.

**Fig. 3:**
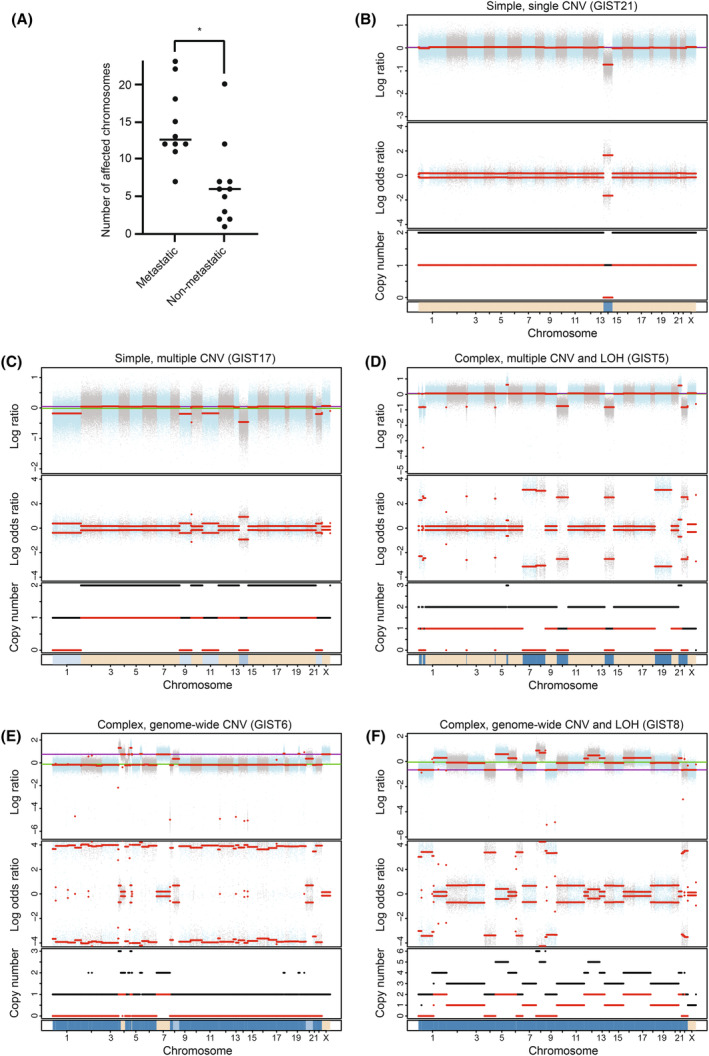
Chromosomal instability in GIST. (A) Level of CIN in the groups of metastatic and non‐metastatic samples, *P‐value = 0.01, Mann–Whitney test. Examples of various patterns of genomic copy number changes and LOH in simple (B and C) and complex (D–F) genomes are shown.

The number of chromosomes affected by CNV and/or copy number neutral LOH was investigated and compared between the two groups. The median number of chromosomes affected was 15 (range 7–23) for the metastatic samples and 6 (range 1–20) for the non‐metastatic group (*P* = 0.01; Fig. 3A). Based on this observation, we further investigated if CIN could be used as a classifier separating the groups. Analysis was performed using the CINmetrics package (https://github.com/lasseignelab/CINmetrics), including the CIN scoring algorithms based on the total number of copy number abnormalities (CNAs or segment breakpoints; [45, 46]), the fraction of the genome altered (fga; [47]), and total aberrations measuring the abundance and genomic size of CNV (tai; [48]) (Data S3). In addition, a CIN score was generated based on the number of chromosomes affected by CNV and LOH. For the various algorithms, individual separation values were determined based on an observed change in the distribution of CIN values. For the algorithm based on the total number of copy number abnormalities, no clear distribution change could be observed. For the total aberration algorithm (tai), most samples clustered on a very narrow distribution with limited possibility to distinguish the samples. When ranking the GIST samples according to the fraction of genome altered (fga) or total number of chromosomes affected (CNV and LOH), the samples clustered in two or more distributions. When considering the clinical behaviour of the patients, the separation obtained from the fraction of the genome altered algorithm could only classify a few metastatic samples as highly complex. However, the complexity score based on the number of chromosomes affected by CNV and/or copy number neutral LOH more clearly separated the metastatic from the non‐metastatic samples according to clinical behaviour (Data S3). Based on the distribution of the number of affected chromosomes (Data S3), we classified samples with 10 or more affected chromosomes as complex, and those with nine or fewer as simple (Table 2).

**Table 2:** Overview of CIN. The number of affected chromosomes per sample includes CNV and/or copy‐neutral LOH. Chr, chromosome.

Sample	Group	# Chr CNV	# Chr LOH	#Chr CNV + LOH	#Chr Total	CIN	Tumour ploidy	Mitotic count
GIST1	Metastatic	8	0	5	13	Complex	1.82	12
GIST3	Metastatic	11	1	0	12	Complex	1.82	35
GIST5	Metastatic	9	3	0	12	Complex	1.91	11
GIST6	Metastatic	18	0	4	22	Complex	1.17	166
GIST7	Metastatic	9	1	2	12	Complex	1.81	20
GIST8	Metastatic	17	1	5	23	Complex	3.27	50
GIST10	Metastatic	10	1	0	11	Complex	1.83	51
GIST11	Metastatic	7	0	0	7	Simple	1.87	3
GIST12	Metastatic	17	0	1	18	Complex	1.72	15
GIST24	Metastatic	13	0	2	15	Complex	1.86	178
GIST13	Non‐metastatic	7	0	0	7	Simple	1.9	11
GIST14	Non‐metastatic	18	0	2	20	Complex	3.47	7
GIST15	Non‐metastatic	3	0	0	3	Simple	1.97	1
GIST16	Non‐metastatic	2	0	0	2	Simple	1.95	7
GIST17	Non‐metastatic	7	0	0	7	Simple	1.90	130
GIST18	Non‐metastatic	5	0	0	5	Simple	1.97	32
GIST19	Non‐metastatic	9	0	3	12	Complex	2.07	13
GIST20	Non‐metastatic	6	0	0	6	Simple	1.85	7
GIST21	Non‐metastatic	1	0	0	1	Simple	1.96	7
GIST22	Non‐metastatic	2	0	0	2	Simple	2.02	2
GIST23	Non‐metastatic	6	0	0	6	Simple	1.75	1

All the samples in the cohort had CNVs, but a large diversity of simple and complex CNV patterns was observed both within and between the GIST groups (Fig. 3B–F, Data S4 and S5). Among the simple samples, the diversity reflected the typical evolution in GIST with an increasing loss of chromosomes (Fig 3B,C). Interestingly, all the 11 complex samples had CNV which caused LOH in several chromosome arms or whole chromosomes (Fig. 3D–F and Table 2). One of the complex metastatic samples showed a loss of one allele in almost all chromosomes (Fig. 3E). An intricate pattern of genome duplication combined with CNV was observed for three complex samples (Fig. 3F). Five of the complex samples (45%) did *not* have a deletion of chromosome 14, a feature regarded as an early event in GIST development and observed to be present in all simple samples.

The presence of high‐confidence, non‐canonical chromothripsis was observed in four out of 10 samples in the metastatic group and only one out of 11 samples in the non‐metastatic group (Table 3). Four out of the five samples with chromothripsis had highly complex genomes. Chromosome 1 and chromosome 19 were the chromosomes most frequently showing a chromotriptic pattern, with three and two of the samples affected, respectively (Table 3).

**Table 3:** Chromothriptic pattern of high‐confidence scored GIST samples. Shown chromosomes with a chromothripsis pattern and the number of copy number switches within 50 Mb for each of the chromosomes.

	Sample group	Chromosomes affected	Switches within 50 Mb	CIN
GIST1	Metastatic	Chr1, Chr3, Chr16, Chr21	9, 12, 14, 10	Complex
GIST7	Metastatic	Chr1	26	Complex
GIST11	Metastatic	Chr8, Chr19	12, 6	Simple
GIST24	Metastatic	Chr7, Chr12, Chr17, Chr19	12, 10, 30, 11	Complex
GIST19	Non‐Metastatic	Chr1, Chr9	31, 8	Complex

### 3.3 The kinetochore and cell cycle signalling are deregulated in high‐risk samples

#### 3.3.1 Deregulation of cell cycle upstream regulators, downstream targets, and cellular functions and pathways are associated with metastasis

Gene expression analysis of the GIST cohort was performed to identify differentially expressed genes between tumours that metastasised and those that did not. Among the 487 differentially expressed genes, 180 were downregulated and 307 were upregulated in metastatic cases (Data S6). Unsupervised hierarchical clustering based on the differentially expressed genes showed good separation of the sample groups (Data S7).

Canonical pathway analysis was performed on the list of differentially expressed genes. We identified 10 canonical pathways that were significantly differentially expressed in metastatic samples compared to non‐metastatic samples. All the pathways were associated with cell cycle regulation, specifically with cell cycle signalling or the kinetochore complex (Table 4). The pathways contained a total of 49 genes (Table 4), of which 47 were upregulated, and only two (*TGFB2* and *CBX72*) were downregulated in metastatic patient samples.

**Table 4:** Significantly activated or inhibited IPA Canonical pathways. Analysis was performed on 487 genes differentially expressed between metastatic and non‐metastatic GIST samples. A negative *z* value connotates an overall pathway's inhibition and a positive *z* value connotates an overall activation, with a cut‐off at |*z*‐score| > 1.6 and Benjamini‐Hochberg adjusted *P* < 0.05.

Ingenuity canonical pathways	Adj *P*‐value	−log(*P*‐value)	Ratio^a^	*z*‐score	Genes
Kinetochore metaphase signalling pathway	6.55E‐15	14.2	0.20	2.52	*AURKB*, *BIRC5*, *BUB1B*, *CCNB1*, *CDC20*, *CDCA8*, *CDK1*, *CENPA*, *CENPU*, *ESPL1*, *H2AX*, *KNL1*, *MXD3*, *NUF2*, *PLK1*, *PTTG1*, *SKA1*, *SKA3*, *SPC24*, *TTK*, *ZWINT*
Mitotic roles of polo‐like kinase	5.08E‐10	9.3	0.20	1.73	*CCNB1*, *CCNB2*, *CDC20*, *CDC25A*, *CDC25B*, *CDK1*, *ESPL1*, *FBXO5*, *KIF11*, *KIF23*, *PKMYT1*, *PLK1*, *PTTG1*
Oestrogen‐mediated S‐phase entry	3.9E‐8	7.41	0.31	2.82	*CCNA2*, *CCNE2*, *CDC25A*, *CDK1*, *E2F1*, *E2F2*, *E2F7*, *E2F8*
Cell cycle: G2/M DNA damage checkpoint regulation	7.14E‐8	7.15	0.20	−1.67	*AURKA*, *CCNB1*, *CCNB2*, *CDC25B*, *CDK1*, *CHEK1*, *CKS2*, *PKMYT1*, *PLK1*, *TOP2A*
Role of CHK proteins in cell cycle checkpoint control	2.63E‐7	6.58	0.18	−1.89	*CDC25A*, *CDK1*, *CHEK1*, *CLSPN*, *E2F1*, *E2F2*, *E2F7*, *E2F8*, *PLK1*, *SLC19A1*
Cyclins and cell cycle regulation	1.25E‐6	5.9	0.13	3.32	*CCNA2*, *CCNB1*, *CCNB2*, *CCNE2*, *CDC25A*, *CDK1*, *E2F1*, *E2F2*, *E2F7*, *E2F8*, *TGFB2*
Cell Cycle: G1/S checkpoint regulation	4.97E‐4	3.3	0.10	−2.45	*CCNE2*, *CDC25A*, *E2F1*, *E2F2*, *E2F7*, *E2F8*, *TGFB2*
Senescence pathway	5.11E‐4	3.29	0.54	−2.32	*ATF3*, *CBX7*, *CCNB1*, *CCNB2*, *CCNE2*, *CDC25A*, *CDC25B*, *CDK1*, *CHEK1*, *E2F1*, *E2F2*, *E2F7*, *E2F8*, *EZH2*, *RASD1*, *TGFB2*
Cell cycle regulation by BTG family proteins	9.89E‐4	3	0.14	2.24	*CCNE2*, *E2F1*, *E2F2*, *E2F7*, *E2F8*
Cell cycle control of chromosomal replication	1.09E‐3	2.96	0.11	2.45	*CDK1*, *CDT1*, *MCM2*, *MCM4*, *ORC1*, *TOP2A*

^a^ A ratio of the number of genes from the gene list that maps to the pathway divided by the total number of genes in IPA that map to the same pathway.

The observed downregulation of *TGFB2* can promote the progression into the late G1 and S phase (reviewed in [49]). Reduced susceptibility to senescence could be promoted by the observed reduced level of the tumour suppressor *CBX7* [50]. Loss of CBX7 is associated with highly malignant phenotypes and poor prognosis in cancer [51] and promotes resistance to TKIs [52]. Among the upregulated genes, there were genes central to the canonical kinetochore complex, such as *CENPA*, *CENPU*, *NUF2*, *KNL1*, *SKA1*, *SKA3* and *ZWINT* and the kinases *AURKA*, *AURKB*, *BUB1B*, *CDK1*, *PLK1* and *TTK* involved in kinetochore assembly regulation and kinetochore attachment. Several of these genes are also central players in cell cycle regulation together with other upregulated genes like the cyclins *CCNB1*, *CCNB2*, *CCNA2* and *CCNE2*, the E2F transcription factors *E2F1*, *E2F2*, *E2F7* and E2F8 and the crucial cell cycle phase regulators *CDC25A* and *CDC25B*. Furthermore, upregulation of the chromosomal replication pathway was observed in the metastatic group. This was evidenced by increased expression of central genes encoding for components of the replication initiation complex, such as *ORC1*, *CDT1* and the *MCM* genes *MCM2* and *MCM4*.

**Fig. 4:**
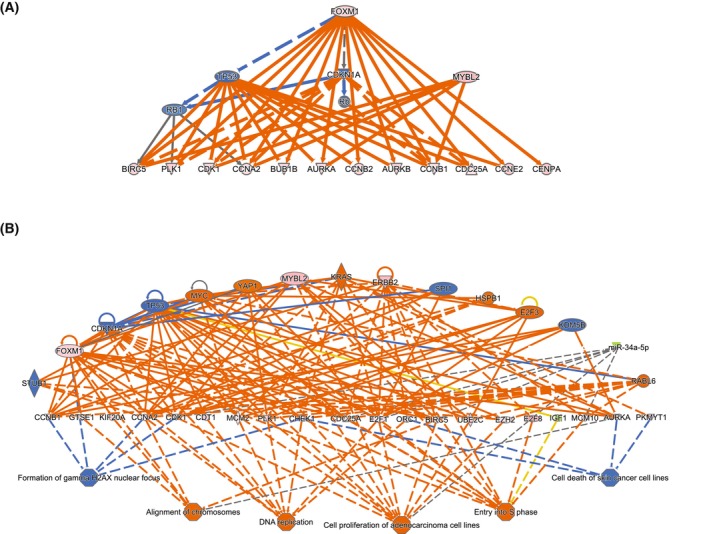
Mechanistic and regulatory networks in GIST. (A) Mechanistic network with FOXM1 and MYBL2 as master upstream regulators. The master regulators mediate the regulation of the target genes through direct regulation or intermediate regulators. (B) Regulatory effects network of upstream regulators, target molecules and the impact on downstream functional mechanisms. Pink: increased gene expression in the dataset. Green: decreased gene expression in the dataset. Blue: predicted inhibition. Orange line: leads to activation. Blue line: leads to inhibition. Grey line: effect not predicted.

An upstream regulator analysis identified potential mechanisms regulating the differentially expressed genes (Data S8). The central upstream regulators *FOXM1*, *MYBL2* (Fig. 4A), *CHAP2L*, *E2F1* and *ERBB2* were differentially upregulated in metastatic samples, and the downstream target genes were observed to be significantly activated. The upstream regulator miR34a was significantly downregulated, and the predicted target genes were upregulated (Fig. 4B). In addition, a set of transcriptional regulators was identified without differential gene expression changes, but the protein was assumed to have changed activity due to the observed activation or inhibition of the downstream target genes (Data S8).

To identify the possible biological effect provided by the differentially expressed genes, we performed a functional analysis in IPA to categorise the differentially expressed genes. Six functional categories were identified; Cell cycle, Cellular assembly and organisation, DNA replication, recombination and repair, Cellular development, cellular growth and proliferation, Cell death and survival and Cellular movement (Table 5). The categories contained a total of 168 unique genes (Data S9). Most of the genes were upregulated (130 genes), with only 36 genes found downregulated in metastatic samples. Interestingly, the strongest downregulated genes were associated with the hormone signalling pathway like somatostatin receptor 1 (*SSTR1*), progesterone receptor (*PGR*), LDL Receptor Related Protein 1B (*LRP1B*) and Insulin‐like growth factor 1 (*IGF1*) where the first three genes are putative tumour suppressors.

**Table 5:** Overview of enriched functional categories identified by IPA analysis of differentially expressed genes between metastatic and non‐metastatic samples. The six main categories are divided further into molecular functions which are predicted to increase or decrease. Cut‐off activation |*z*‐score| > 2, B‐H corrected *P*‐value<0.05.

Categories	Functions^a^	*P*‐value	Predicted activation state	Activation *z*‐score
Cell cycle	Mitosis	2.90E‐21	Increased	2.417
	M phase of tumour cell lines	2.31E‐07	Increased	2.056
	S phase	9.78E‐07	Increased	2.063
	Interphase	2.84E‐06	Increased	2.220
Cellular assembly and organisation	Alignment of chromosomes	8.01E‐10	Increased	2.345
	Formation of gamma H2AX nuclear focus	5.84E‐04	−2.376	−2.376
DNA replication, recombination, and repair	DNA replication	2.34E‐05	Increased	2.470
	Repair of DNA	9.88E‐05	Increased	2.142
	Metabolism of DNA	5.38E‐04	Increased	2.110
Cellular development, cellular growth and proliferation	Cell proliferation of carcinoma cell lines	5.62E‐08	Increased	3.120
Cell death and survival	Cell viability	3.59E‐05	Increased	3.730
	Cell death of carcinoma cell lines	3.54E‐04	Decreased	−2.596
Cellular movement	Invasion of cells	1.06E‐02	Increased	2.034

^a^ Included each main function from the analysis only once.

A regulatory effect analysis provided a larger combined overview of the signalling networks suggested to be deregulated when comparing the high‐risk GIST groups. The networks illustrate how the activated or inhibited upstream regulators can affect the downstream target molecules with the given impact on downstream molecular functions. One of the larger networks is shown in Fig. 4B, highlighting the upstream regulators CDKN1A, E2F3, ERBB2, FOXM1, HSPB1, KDM5B, KRAS, miR‐34a‐5p, MYBL2, MYC, RABL6, SPI1, STUB1, TP53 and YAP1. Of these, *MYC* was found to be recurrently gained among metastatic samples. The upstream regulators in Fig. 4B were predicted to affect target molecules involved in cell cycle regulation, like cyclins and kinases, which are central players in DNA replication and repair, cell death and proliferation. To further investigate the observed increase in proliferation genes, GSEA was run using a cell proliferation gene set. A gene expression enrichment for cell proliferation was seen in the metastatic group (*P*‐adjusted = 0.003, ES = 0.345). This is further supported by the observed differences in the number of mitoses per 50 high‐power fields of view, with a median of 28 for the metastatic group and 7 for the non‐metastatic (Table 1).

#### 3.3.2 Integration of miRNA and mRNA data identifies interaction networks associated with the cell cycle

MicroRNA gene expression analysis identified 25 miRNAs to be differentially expressed between the two groups of high‐risk tumours. The only upregulated miRNAs in metastatic samples were miR‐196b‐5p, miR‐194‐5p and miR‐424‐5p, and the most downregulated miRNAs were miR‐514a‐3p, miR‐497‐5p and miR‐218‐5p (Data S10). Hierarchical clustering using the differentially expressed miRNAs separated the GIST samples into two main clusters, one dominated by metastatic and the other by non‐metastatic samples (Data S11).

**Fig. 5:**
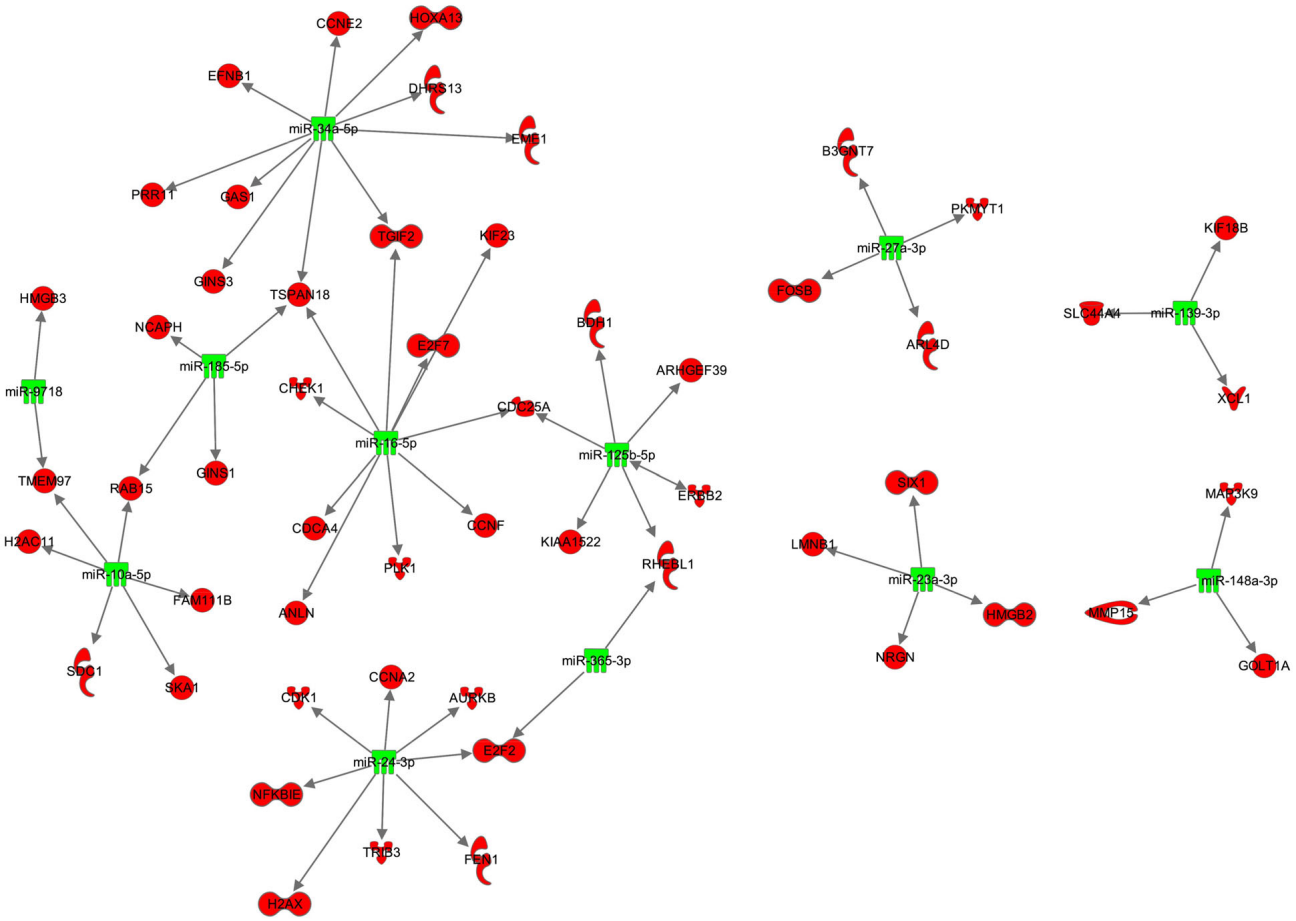
Interaction networks between miRNAs and mRNAs. Shown networks where both the miRNAs and mRNAs are differentially expressed, and the mRNAs are predicted target genes with opposite expression levels of the miRNAs. Green downregulated miRNA, and red upregulated mRNA.

The interaction between the differentially expressed miRNAs and mRNAs was predicted using the microRNA target filter in IPA with a confidence level set to experimentally observed and/or highly predicted interactions. Of the 25 differentially expressed miRNAs, 20 were predicted to directly target 63 of the differentially expressed mRNAs (Data S10). As specified, all the miRNA and mRNA pairs had opposite fold changes; all the miRNAs were observed to be downregulated in metastatic samples, and the target mRNAs were upregulated. The individual miRNAs had up to 10 predicted mRNA targets, of which the largest networks are shown in Fig. 5. Of the 63 mRNAs predicted to be regulated by the miRNAs, 20 were associated with cell cycle regulation and the kinetochore, including the genes *AURKB*, *CCNA2*, *CDC25A*, *CDK1*, *E2F2*, *E2F7*, *H2AX*, *KIF23* and *PLK1* (Data S10), supporting a post‐transcriptional regulation.

## 4 Discussion

Here, we have used a multi‐omics approach to analyse a cohort of patients with high‐risk gastric GISTs. We have compared genomic and transcriptomic tumour profiles from patients who developed metastasis to patients without metastasis after long‐term follow‐up. Our results show intrinsic differences in gene expression and DNA copy number changes, providing evidence of distinct biology among high‐risk GISTs with different metastatic potential.

The mutational burden was low in GISTs, even for the most aggressive subgroup of patients, which is consistent with previously published data [16]. No recurrent actionable somatic mutations were expressed except for mutations in *KIT* and *PDGFRA*. Interestingly, only a low fraction (~ 30%) of the somatic mutations were found to be expressed, which is in line with the observations previously seen in colorectal cancer [53], and lower when compared to prostate cancer [54]. This has a big impact on the interpretation of the actionability of somatic mutations in cancer without knowledge regarding the expression of the mutated allele.

Chromosomal instability was the most pronounced genomic feature, and we observed both CNVs and copy number neutral LOH. Most metastasizing high‐risk GISTs showed complex CIN profiles, while the less aggressive, non‐metastatic tumours showed a simpler CIN pattern. Loss of chromosome 14 is the earliest and most frequent aberration in GIST, followed by loss of additional chromosomes in higher‐risk tumours [55, 56, 57]. Based on the pattern of CNVs, the groups in our cohort seem to represent different stages of the genomic evolution of GIST, as the simplest samples contain only a deletion of one copy of chromosome 14, in addition to a *KIT* or *PDGFRA* mutation. Our finding is in line with the general assumption that CIN fosters much of the intratumoural heterogeneity observed in cancers and drives phenotypic variation facilitating adaptation during tumour evolution.

Genomic complexity involving copy‐neutral LOH of whole chromosomes or chromosome arms has been reported as a frequent event in GIST [58]. Copy‐neutral LOH can be due to a loss of chromosome material during abnormal mitosis, followed by duplication of the remaining material. This may contribute to the multi‐hit inactivation of tumour suppressors. The presence of chromothripsis was observed more frequently in high‐risk GIST samples with a more aggressive phenotype. A few cases of chromothripsis in GIST have been observed in a pan‐cancer study describing the landscape of chromothripsis [36].

Chibon and co‐workers have demonstrated that a high number of CNVs is associated with inferior outcomes in AFIP intermediate‐risk GISTs [8, 9], indicating that such patients might benefit from adjuvant imatinib treatment. On the other hand, approximately half of the patients classified as mNIH high‐risk may be cured by surgery alone [5]. Thus, identifying prognostic biomarkers within the mNIH high‐risk group could lead to an improved selection of patients for adjuvant therapy and ultimately spare high‐risk patients with a good prognosis for the toxicity and cost of three years of imatinib treatment. In a recent study by Boye et al., [10] karyotyping was used to detect chromosomal aberrations in 206 tumours, of which 76 had a complex karyotype with > 5 chromosomal aberrations. High‐risk patients with a simple tumour karyotype had an estimated 5‐year RFS of 94%, while patients with a complex karyotype had 51%. These studies strongly support further investigation of CIN as a prognostic biomarker to improve patient selection for adjuvant treatment, and the present study provides a detailed genomic and transcriptomic landscape to increase our understanding of high‐risk GISTs.

When comparing expression differences between the two groups of high‐risk tumours, we see a deregulated gene expression signature of proteins involved in all phases of the cell cycle. This was inhibition of G1/S and G2/M checkpoint pathways, reduced senescence and increased chromosomal replication in aggressive high‐risk tumours. The upregulated *CDK1* is a central player with a coordinating role in both cell cycle regulation and DNA replication [59]. The pathway inhibition of the G2/M checkpoints, through increased levels of *AURKA*, *CDK1*, and *CCNB1/2*, can facilitate cells with damaged DNA to enter into the M phase, causing increased genomic instability. As an upstream regulator, the transcription factor MYBL2 is involved in cell survival, proliferation and differentiation, and transactivates late cell cycle genes in the G2/M phase. The increased MYBL2 gene expression in metastatic samples is in line with an observed overexpression and poor patient outcomes in numerous cancer entities (reviewed in [60]). *ERBB2* is another upstream regulator observed to be upregulated in the metastatic samples and is a major driver of cell proliferation and angiogenesis. In a previous study, ERBB2 protein (Her2/neu) expression has been significantly correlated with risk grade, tumour size, mitotic count, and increased risk of relapse in primary GIST [61]. Overexpression of the upstream regulator MYC has previously been shown to reversibly induce and maintain CIN, contributing to aneuploidy, tumorigenesis, and tumour evolution [62].

In addition to a deregulated cell cycle, an enrichment in cell proliferation was seen for the metastatic samples. The enhanced proliferation capacity can be seen as a result of adaptation to elevated CIN. However, it is difficult to conclude if the deregulated gene expression is a consequence of CIN or facilitates CIN or both. The possible mechanisms driving CIN are diverse, including mitotic defects, defects in DNA replication and aneuploidy‐driven CIN (reviewed in [63]). In particular, the dysfunctional control of the kinetochore, which can induce mistakes in the alignment and separation of the sister chromatids during the cell cycle, can represent a possible source for chromosome instability in GIST, as seen for other cancers (reviewed in [64]). A strong enrichment for the kinetochore complex and mitotic spindle organisation genes in the metastatic samples was observed. Several of the upregulated core kinetochore genes identified in our study have previously been seen to be periodically and coordinately expressed during the cell cycle [65], and their proteins temporarily localise to kinetochores only during mitosis. In addition to this broad cell division program, additional genes are essential for cell cycle progression and DNA replication was upregulated. Of these, the aurora kinases *AURKA* and *AURKB* have been shown to enhance the generation of aneuploid cells, facilitating genomic instability and malignant transformation (reviewed in [66]). Overexpression of *AURKA* has been suggested to be an independent unfavourable prognostic factor in both treatment‐naïve [21, 67] and imatinib‐treated advanced GIST patients [68]. AURKA overexpression has been shown to enhance the resistance of GIST cells to imatinib [21], thus an AURKA inhibitor may have potential as a therapeutic agent for both imatinib‐sensitive and imatinib‐resistant GIST [68].

The upregulation of the kinetochore genes, including AURKA and AURKB, as well as other cell cycle genes can be facilitated by the transcription factor FoxM1 [65, 69, 70], which shows a strong upregulation in our GIST samples that developed metastasis. FoxM1 binding is significantly enriched at the G2/M phase of the cell cycle, and strong control is required for the proper execution of the mitotic program and to maintain chromosome stability [71]. Increased expression of FOXM1 is observed in a variety of cancer types, and elevated expression has been related to poor overall survival [72].

Different transcriptional miRNA profiles between the two groups of high‐risk patients were identified, with a strong association with the cell cycle. The miRNA miR‐34a‐5p, seen to be downregulated in the metastatic sample group in our study, was also predicted to be an important upstream regulator. miR‐34a‐5p was inversely expressed to a high number of target mRNAs in our datasets, including the G1/S‐specific cyclin *CCNE2*. miR‐34a is located in the chromosome 1p region, which is recurrently deleted in the metastatic group. In a study comparing miRNA and mRNA expression profiles of imatinib‐naïve and imatinib‐resistant primary GIST, analyses revealed a deregulation of cell cycle progression and cellular proliferation with an increased expression of miR‐34a‐5p in the imatinib‐resistant group [15]. The miR‐34 family is a direct target of TP53, and when up‐regulated, it induces apoptosis, cell cycle arrest, and senescence in cancer (reviewed in [73]). The observed downregulation of miR‐218‐5p was in line with previous studies observing miR‐218‐5p to be the most decreased miRNA in GIST compared to normal gastric tissue [74] and also separating high‐risk GIST from low and intermediate‐risk samples [75]. miR‐218 has been shown to regulate KIT protein expression and inhibit GIST cell proliferation and invasion *in vitro* [76]. miR‐218 overexpression might improve the sensitivity of GIST cells to imatinib through PI3K/AKT signalling pathway [77].

Several studies have revealed the enrichment of deregulated cell cycle processes in GIST [14, 15, 16, 17, 18, 19, 20, 21]. Using a cohort comparable to ours, including only primary, treatment‐naïve, high‐risk GIST, samples with higher mitotic counts were shown to have a significantly increased protein expression of cyclin D1, p53 and E2F1 [17]. This associated cell cycle deregulation with a more aggressive phenotype, in line with our results. However, most studies include heterogeneous groups both when it comes to risk classification and treatment status. By comparing gene expression of primary, imatinib‐naïve GIST with imatinib‐resistant, an enrichment for regulators of oestrogen‐mediated S‐phase entry, cyclins and cell cycle regulators, as well as G2/M checkpoint regulation pathways, was seen [15]. Enrichment of the cell cycle has also been identified comparing metastatic and high‐risk GIST to less aggressive GIST [16]. However, these previous studies have consisted of more heterogeneous patient cohorts, and it is difficult to separate intrinsic tumour biology from the effects of TKI treatment.

## 5 Conclusion

Patients with high‐risk GIST are clinically challenging as our biological understanding of the clinical heterogeneity is limited. And there are no available biomarkers able to reliably predict which patients will later develop metastatic disease. Our multi‐omics approach shows that the presence of complex CIN and deregulated cell cycle processes were the most prominent features in this group. Our findings indicate that the increased CIN in the tumours with higher metastatic capacity could be a phenotype of the deregulated cell cycle, including dysfunctional control of the kinetochore machinery. We show that complex CIN and cell cycle control are intrinsically deregulated processes associated with metastasis in gastric, high‐risk GIST. Our results suggest that CIN should be further investigated as a prognostic biomarker in high‐risk GIST. We further show that an improved understanding of the genomic and transcriptomic changes in tumours from a carefully selected patient cohort may inform the development of clinically useful biomarkers.

## Conflict of interest

The authors declare no conflict of interest.

## Author contributions

HMN performed experiments, data interpretation, and study design and drafted the manuscript. KK performed bioinformatics analysis, and data interpretation and drafted the manuscript. SN and DV performed bioinformatics analysis for variant interpretation. EH conceived of bioinformatics analysis. OM performed project planning, design and grant application. KB performed the study design, provided clinical samples and conceived the study. LAM‐Z performed data interpretation, study design and conceived of the study. All authors read and approved the final manuscript.

### Peer review

The peer review history for this article is available at https://www.webofscience.com/api/gateway/wos/peer‐review/10.1002/1878‐0261.13514.

## Supporting information


**Data S1.** Variant calling files were processed and annotated using PCGR.

**Data S2.** Genes located in recurrent focal and arm‐level copy number regions.

**Data S3.** A genomic complexity score analysis for the GIST samples.

**Data S4.** Allele‐specific DNA copy‐number variation and frequency analysis outputs were generated using FACETS.

**Data S5.** Plots showing copy‐number, LOH and ploidy for each of the GIST samples generated using FACETS.

**Data S6.** Normalised expression data of 487 differentially expressed genes.

**Data S7.** Hierarchical clustering of GIST based on mRNA expression.

**Data S8.** IPA Upstream Regulatory Analysis and Functional Categories for the differentially expressed genes.

**Data S9.** IPA Functional categories for the differentially expressed genes.

**Data S10.** Normalised miRNA expression data of 25 differentially expressed miRNAs and overview of predicted mRNA interactions.

**Data S11.** Hierarchical clustering analysis of miRNA expression.

## Data Availability

For the miRNA Nanostring experiments, MIAME‐compliant data that support the findings in this study are openly available from the GEO data repository (www.ncbi.nlm.nih.gov/geo/) under the accession number GSE225978. The exome and RNA‐Seq data of this study are available on request from the corresponding author. The data are not publicly available due to privacy or ethical restrictions.
