# Supplementary material for: Chromosomal instability and a deregulated cell cycle are intrinsic features of high‐risk gastrointestinal stromal tumours with a metastatic potential
Source: Mol Oncol. 2023 Sep 3;17(11):2432–50. doi: 10.1002/1878-0261.13514 (PMC10620130; doi:10.1002/1878-0261.13514)
Supplement: Supplementary file 5 — Data S5. Plots showing copy‐number, LOH and ploidy for each of the GIST samples generated using FACETS. [file MOL2-17-2432-s001.pdf]

GIST1

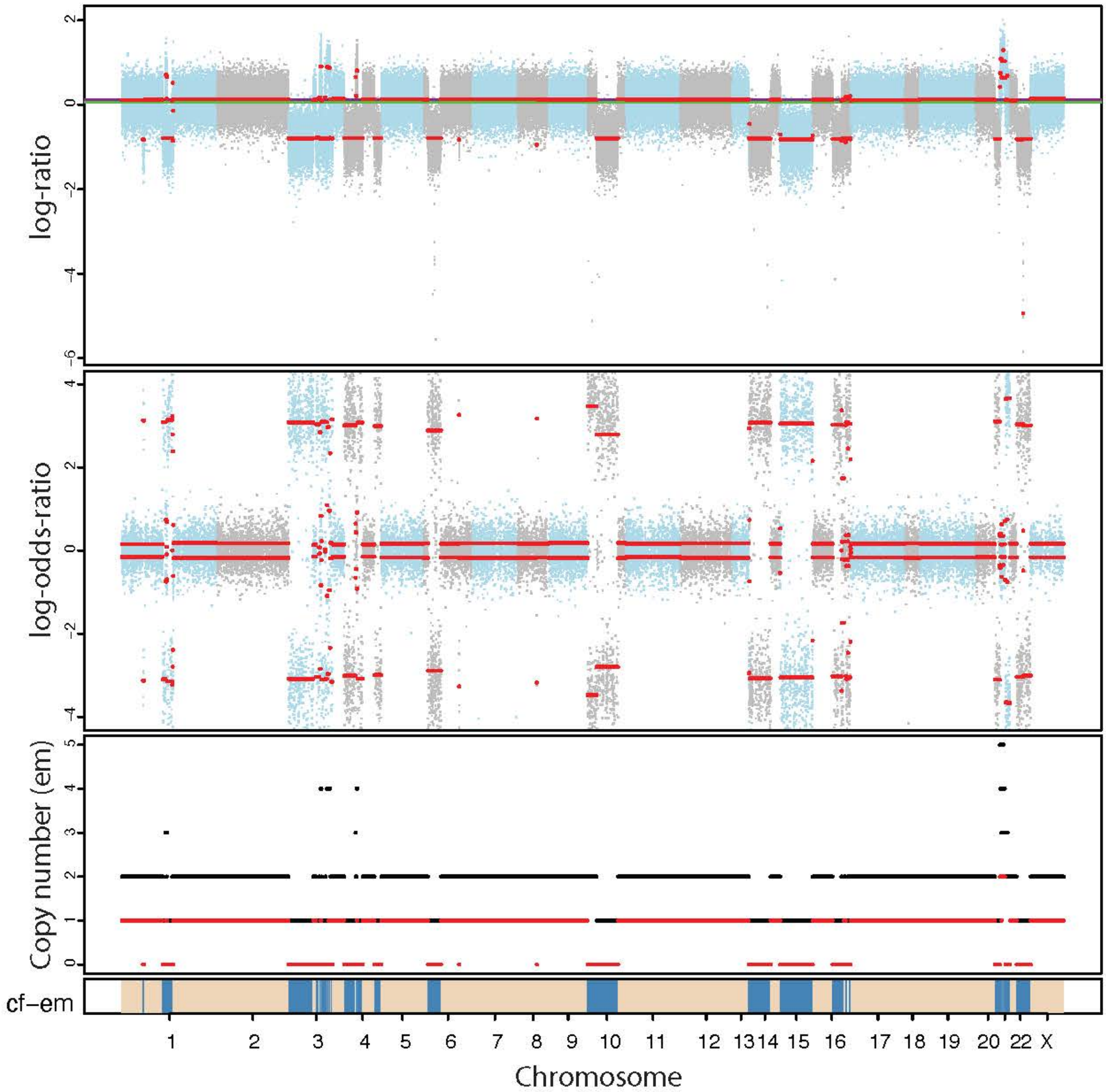

[Purity: 0.95, Ploidy: 1.82, Diploid LogR: 0.13, Log likelihood: 199.15]

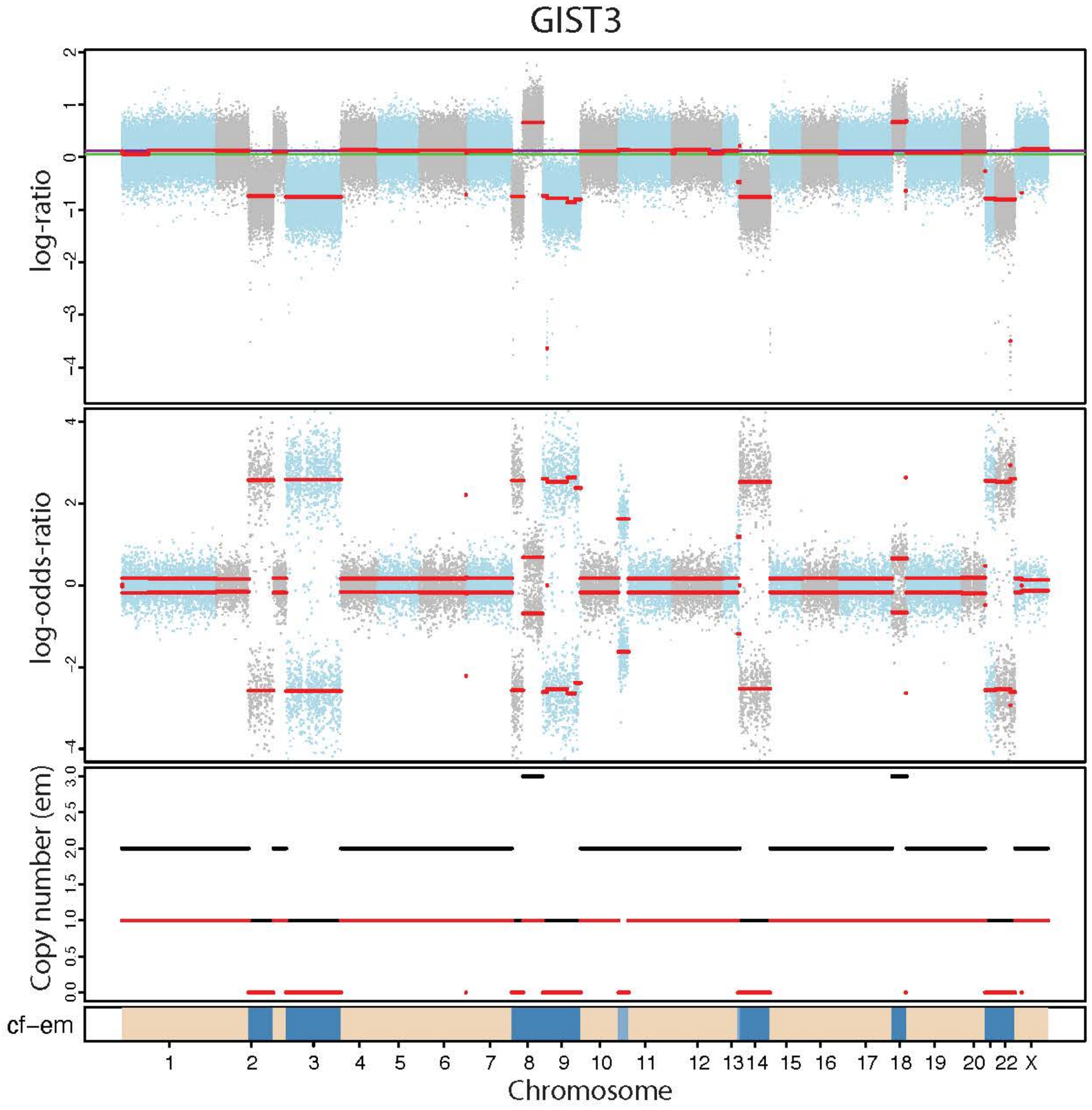

[Purity: 0.92, Ploidy: 1.82, Diploid LogR: 0.13, Log likelihood: 107.65]

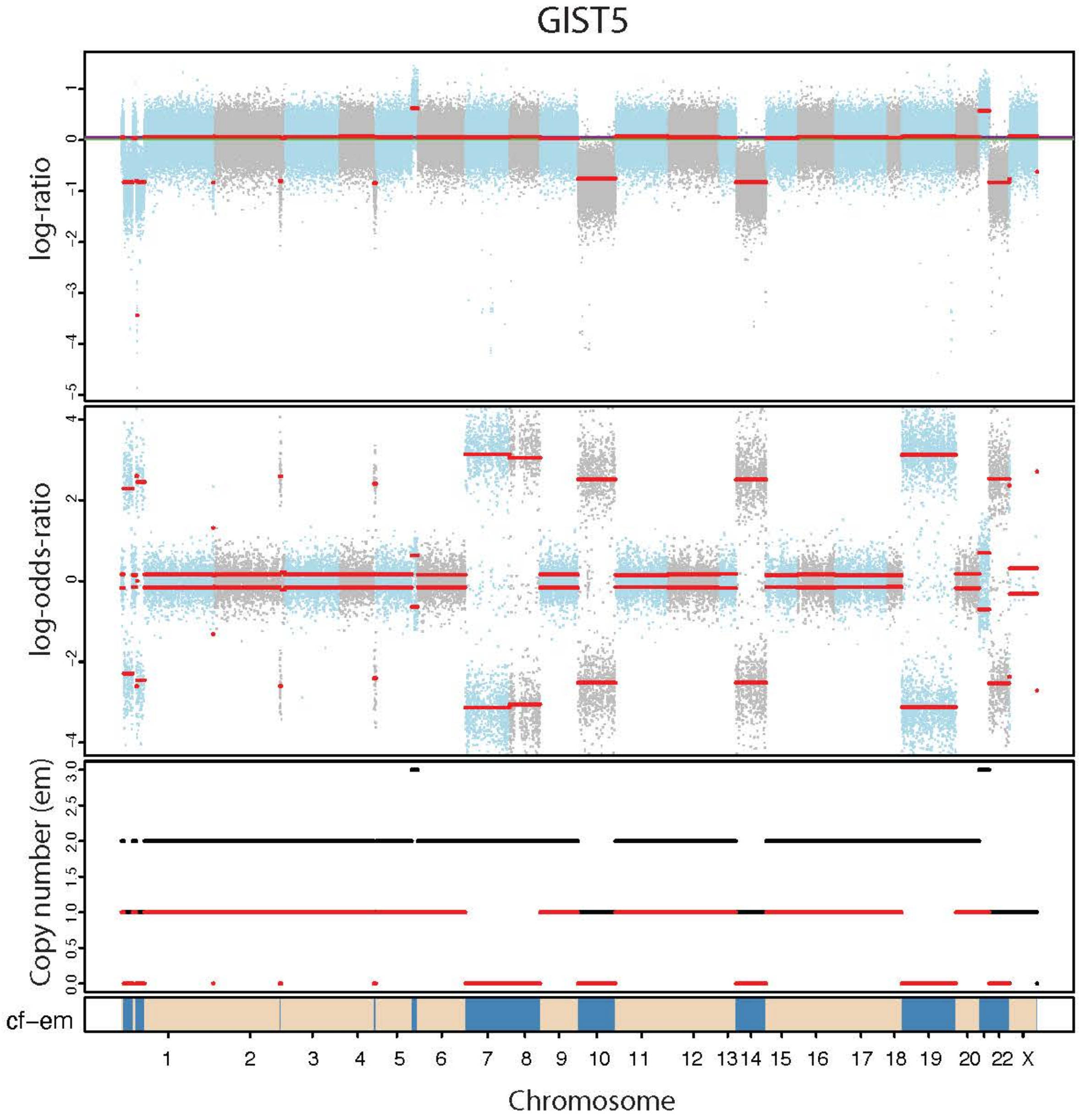

[Purity: 0.92, Ploidy: 1.91, Diploid LogR: 0.06, Log likelihood: 96.3]

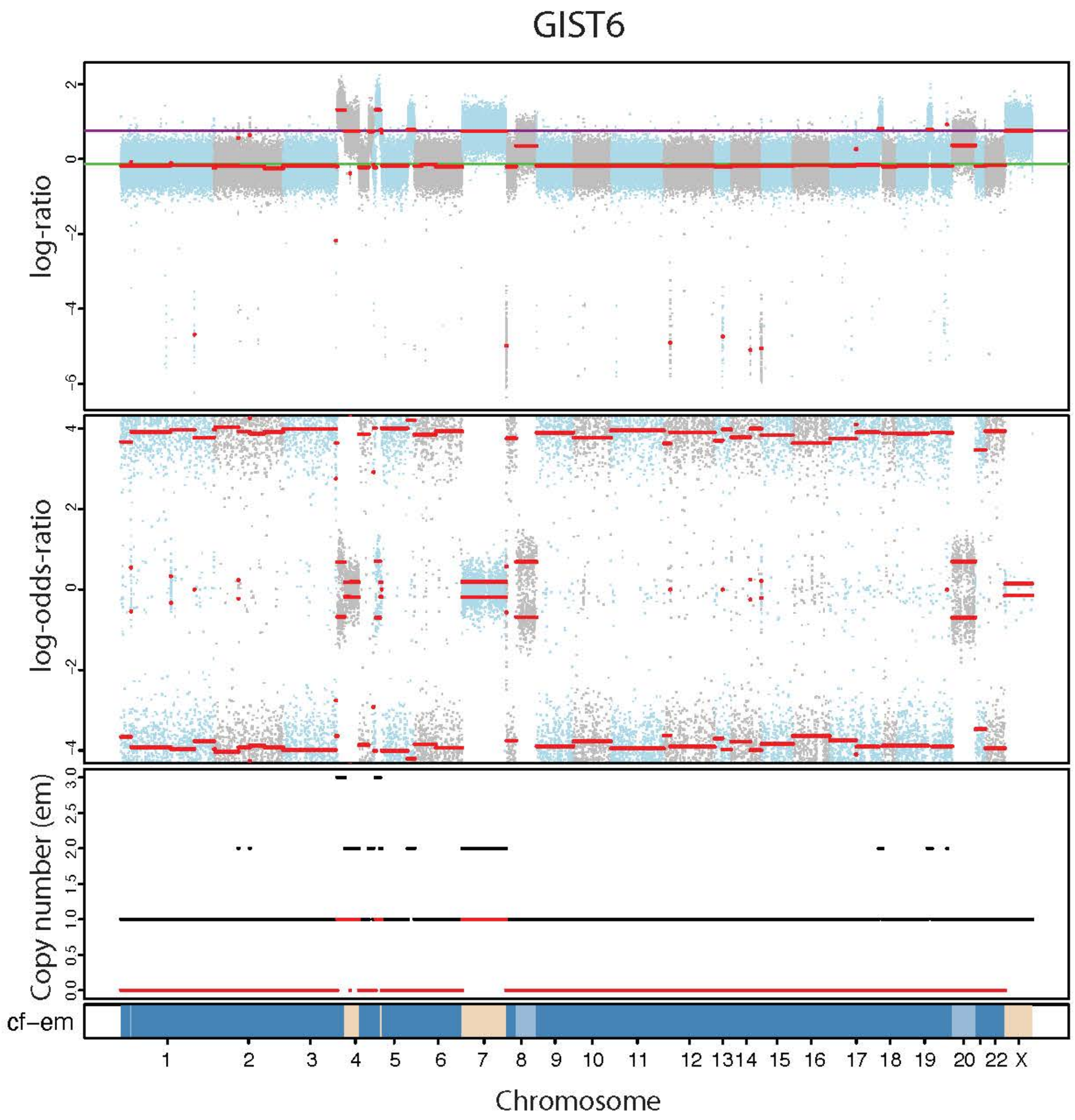

[Purity: 0.98, Ploidy: 1.17, Diploid LogR: 0.75, Log likelihood: 159.94]

GIST7

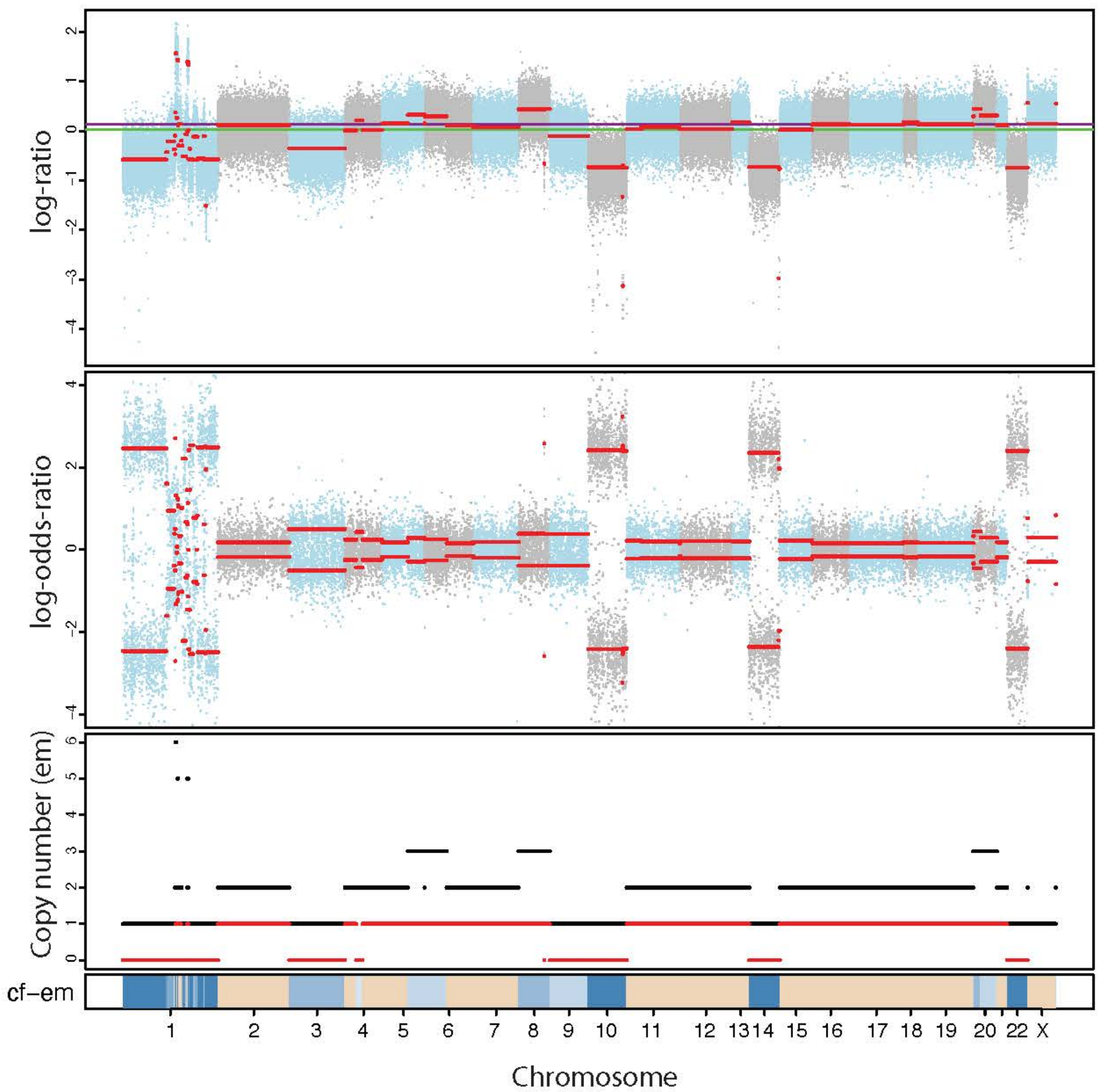

[Purity: 0.9, Ploidy: 1.81, Diploid LogR: 0.13, Log likelihood: 336.07]

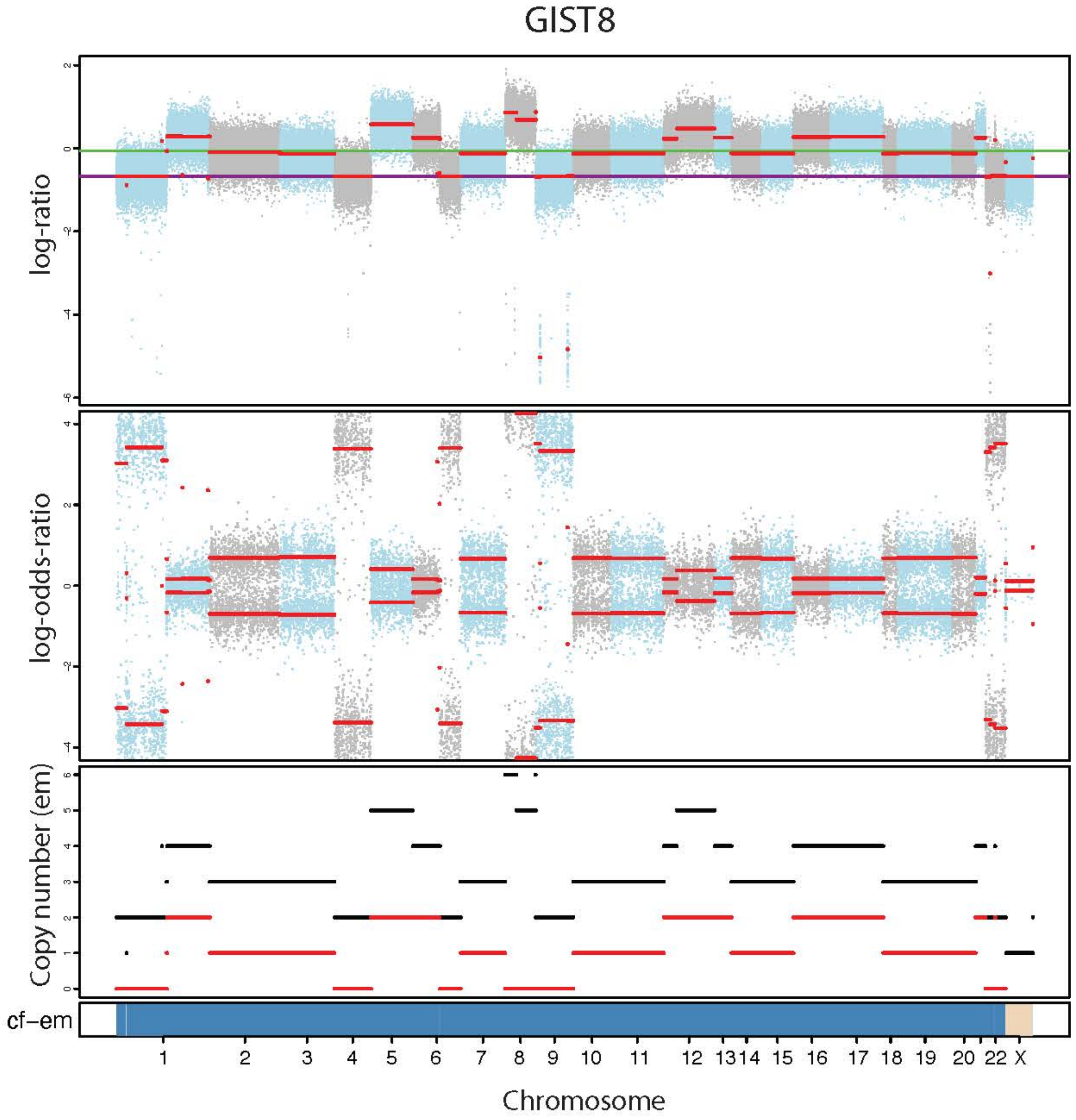

[Purity: 0.94, Ploidy: 3.27, Diploid LogR: −0.67, Log likelihood: 175.07]

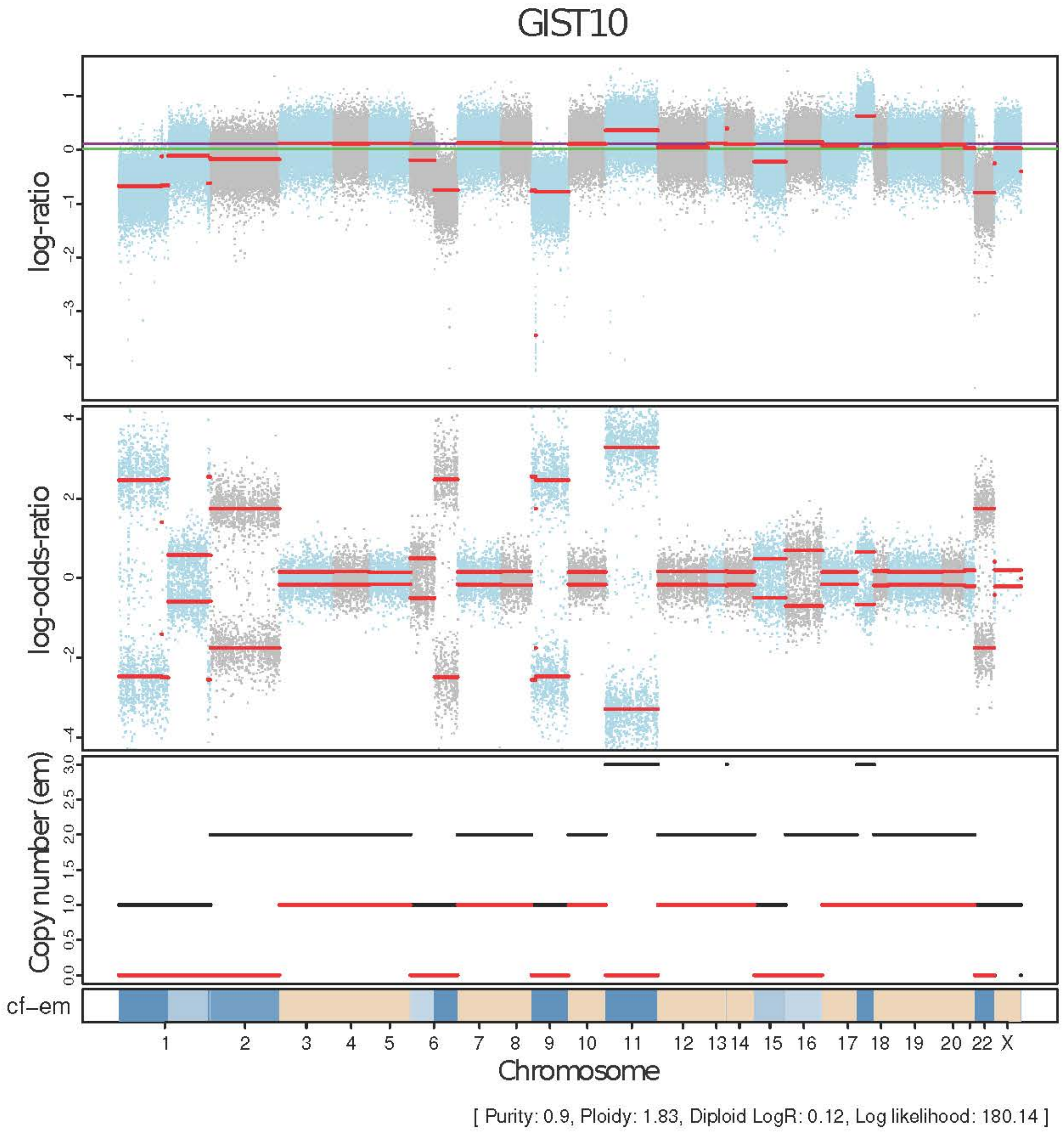
GIST10
log-ratio
log-odds-ratio
Copy number (em)
cf−em
Chromosome
1
2
3
4
5
6
7
8
9
10
11
12
13
14
15
16
17
18
19
20
22
X
[Purity: 0.9, Ploidy: 1.83, Diploid LogR: 0.12, Log likelihood: 180.14]

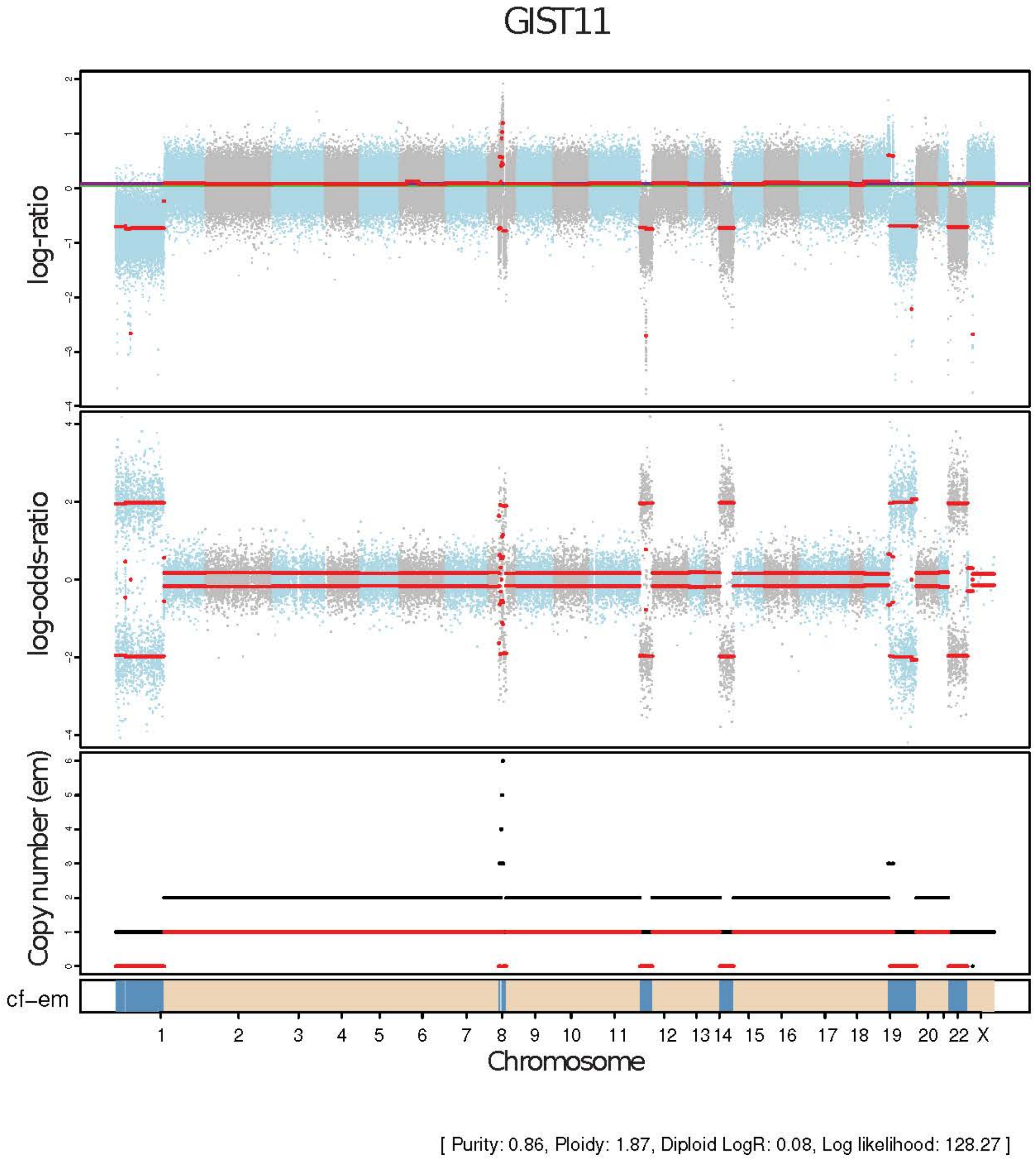
GIST11
log-ratio
log-odds-ratio
Copy number (em)
cf−em
Chromosome
1 2 3 4 5 6 7 8 9 10 11 12 13 14 15 16 17 18 19 20 22 X
[Purity: 0.86, Ploidy: 1.87, Diploid LogR: 0.08, Log likelihood: 128.27]

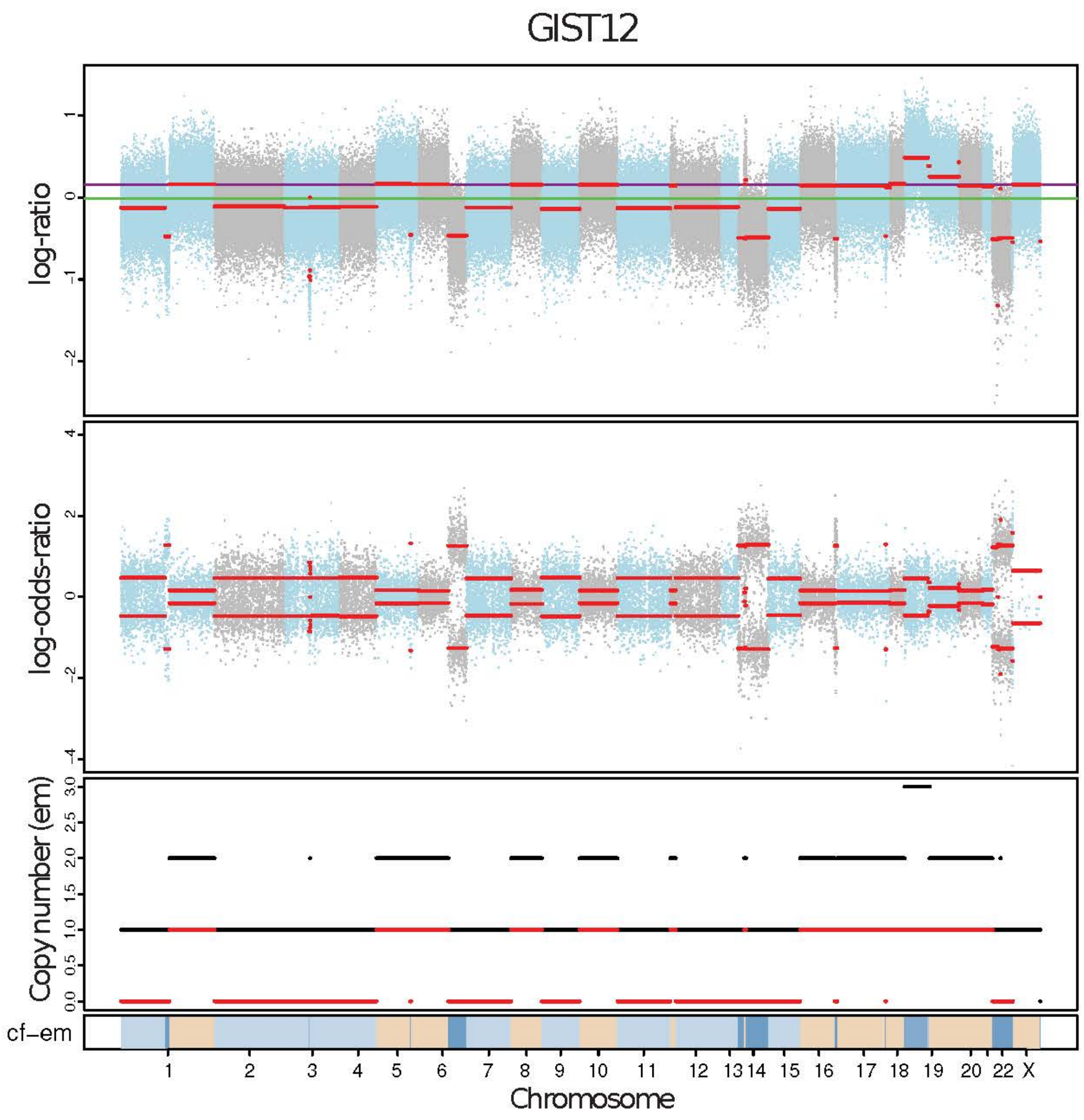

[Purity: 0.72, Ploidy: 1.72, Diploid LogR: 0.15, Log likelihood: 126.76]

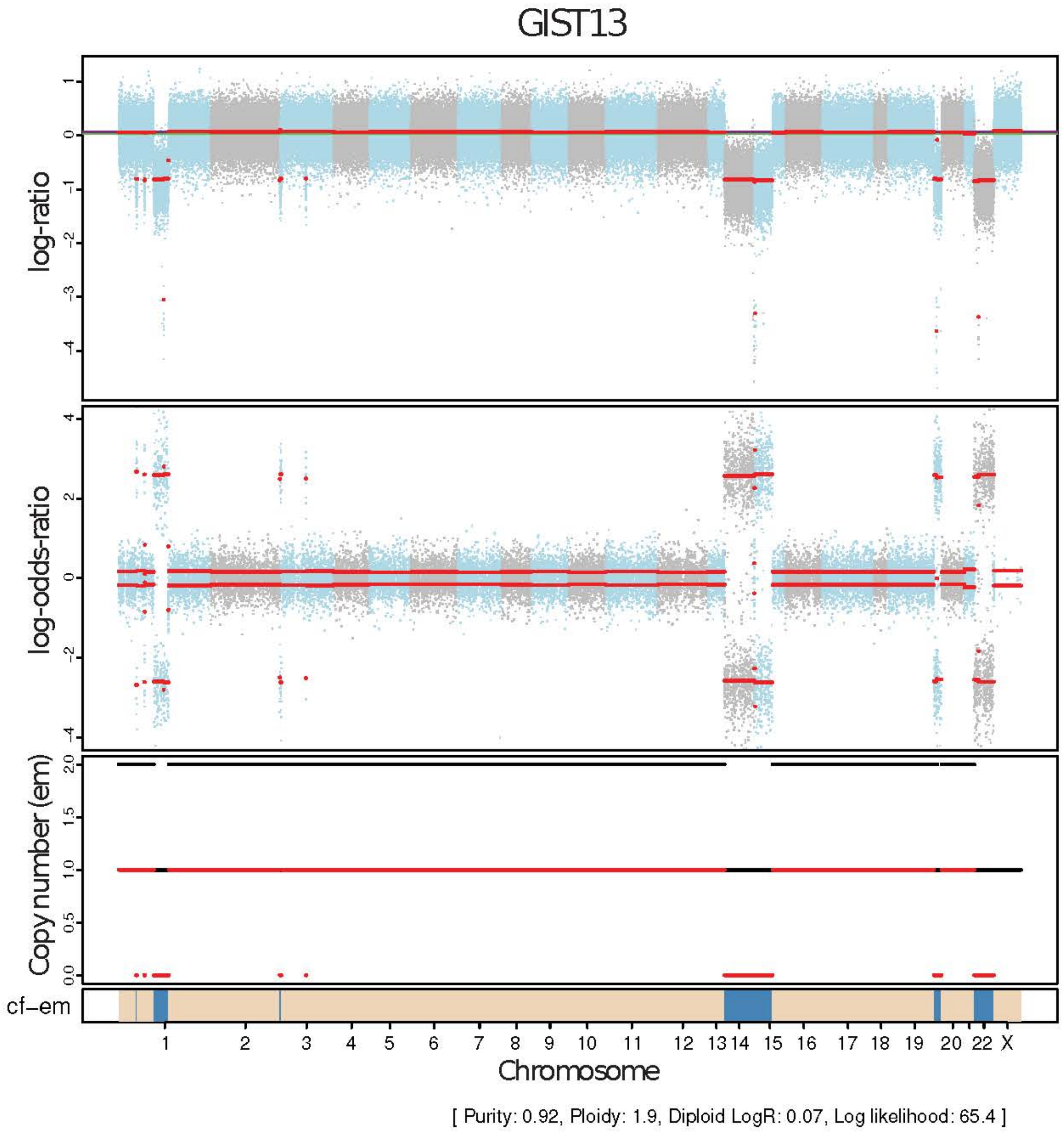
GIST13
log-ratio
log-odds-ratio
Copy number (em)
cf−em
1
2
3
4
5
6
7
8
9
10
11
12
13
14
15
16
17
18
19
20
22
X
Chromosome
[Purity: 0.92, Ploidy: 1.9, Diploid LogR: 0.07, Log likelihood: 65.4]

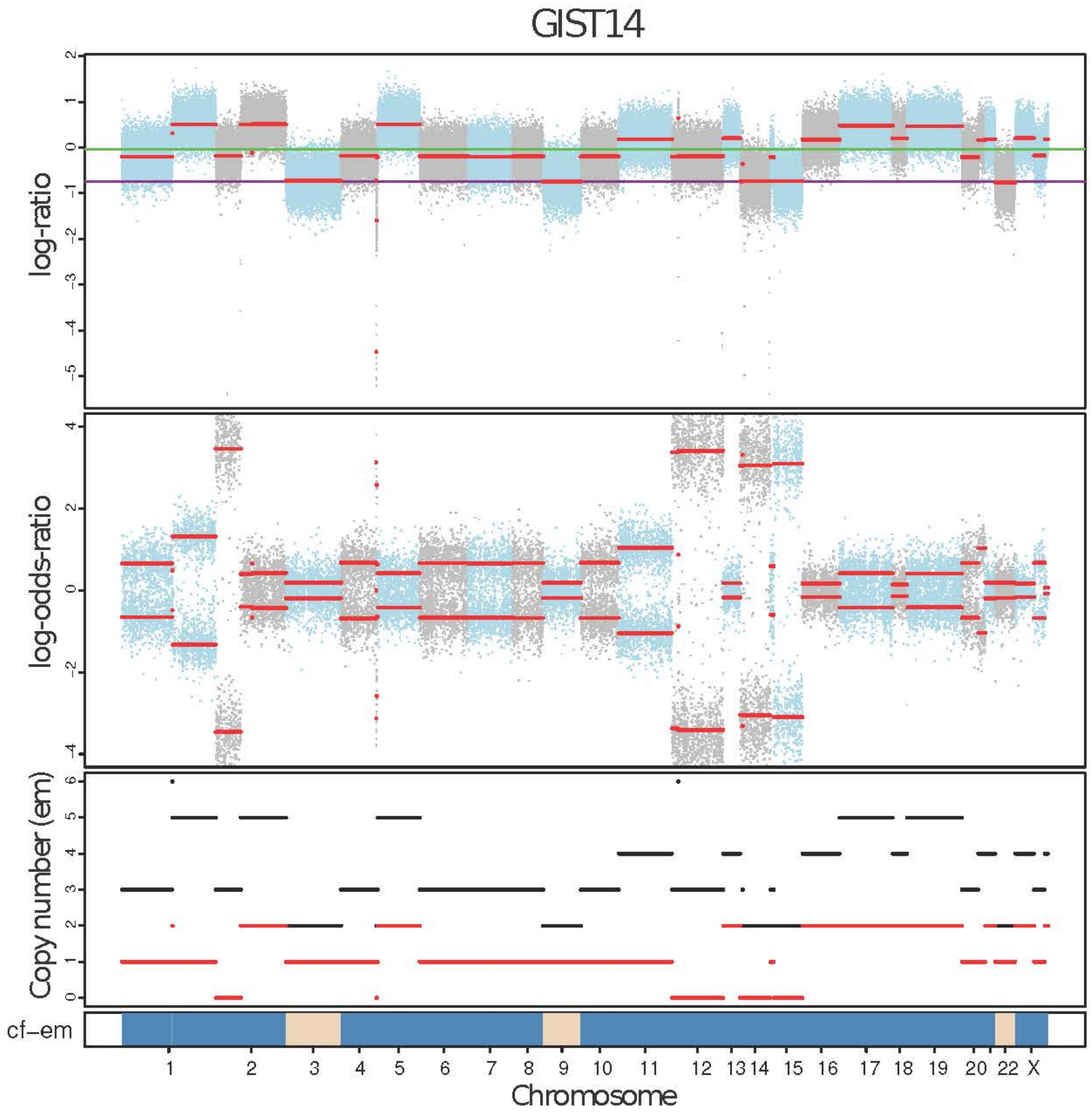

[Purity: 0.91, Ploidy: 3.47, Diploid LogR: −0.74, Log likelihood: 142.42]

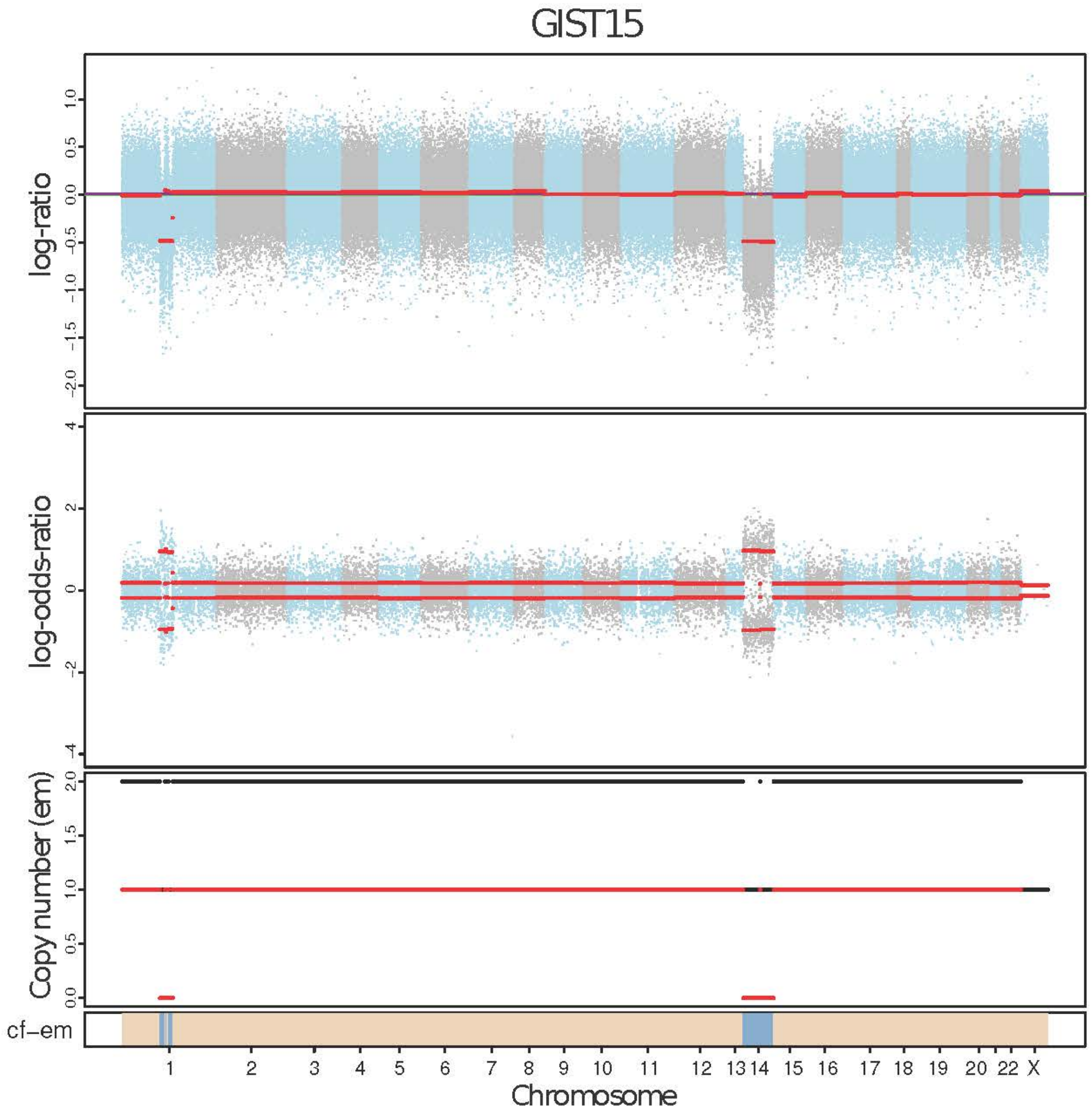

[Purity: 0.61, Ploidy: 1.97, Diploid LogR: 0.01, Log likelihood: 47.03]

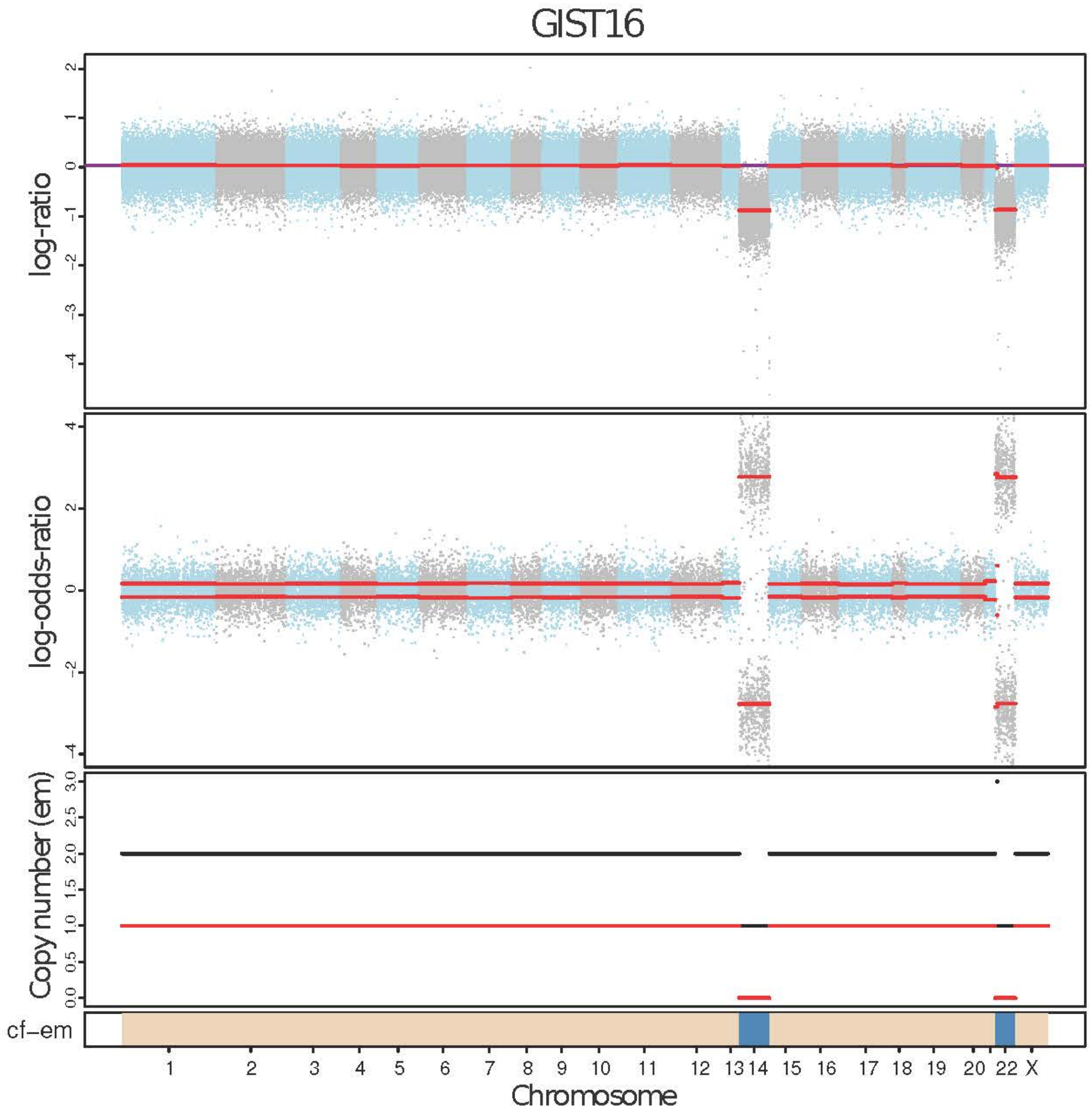

[Purity: 0.94, Ploidy: 1.95, Diploid LogR: 0.04, Log likelihood: 29.92]

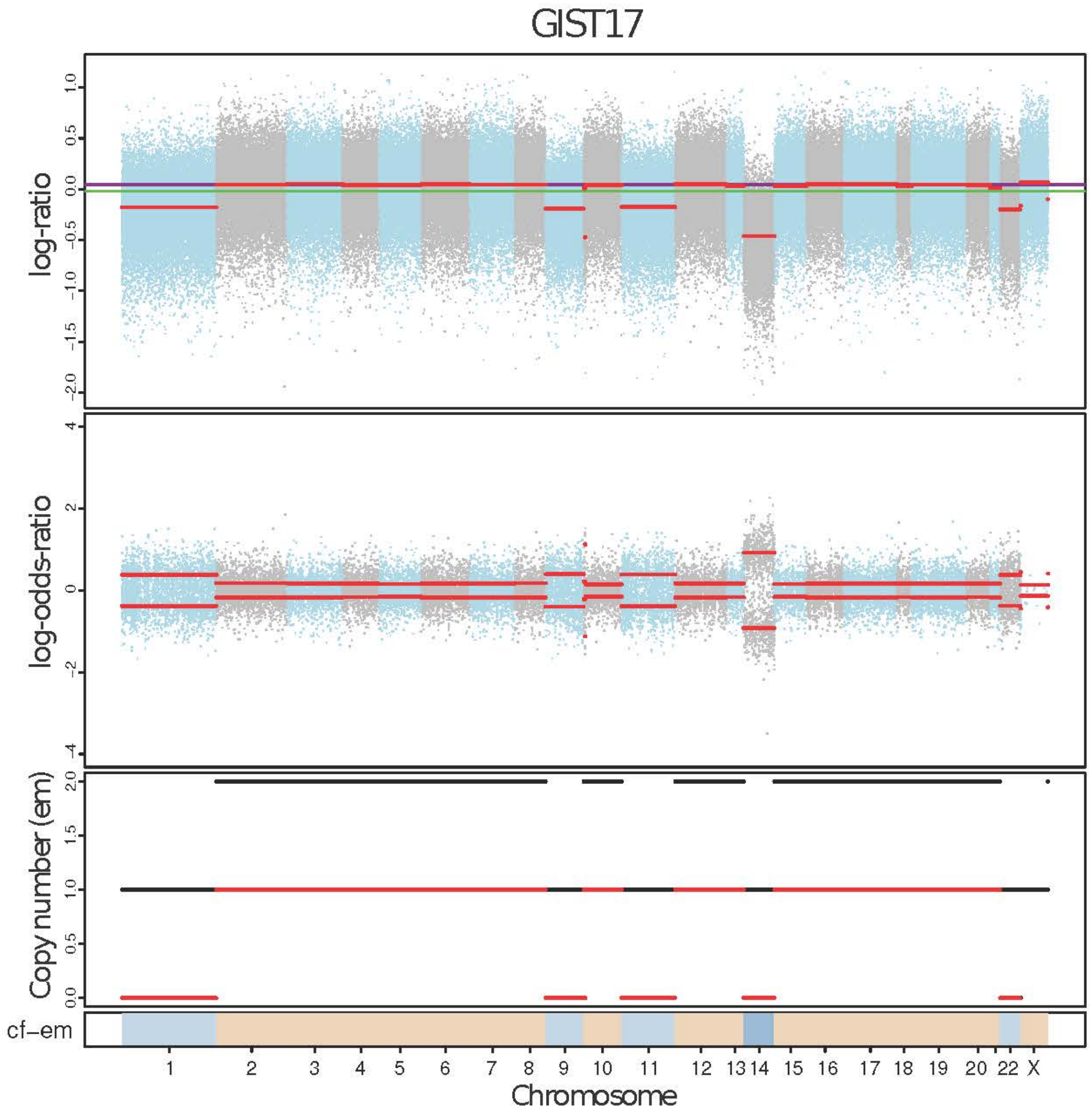

[Purity: 0.6, Ploidy: 1.9, Diploid LogR: 0.04, Log likelihood: 55.88]

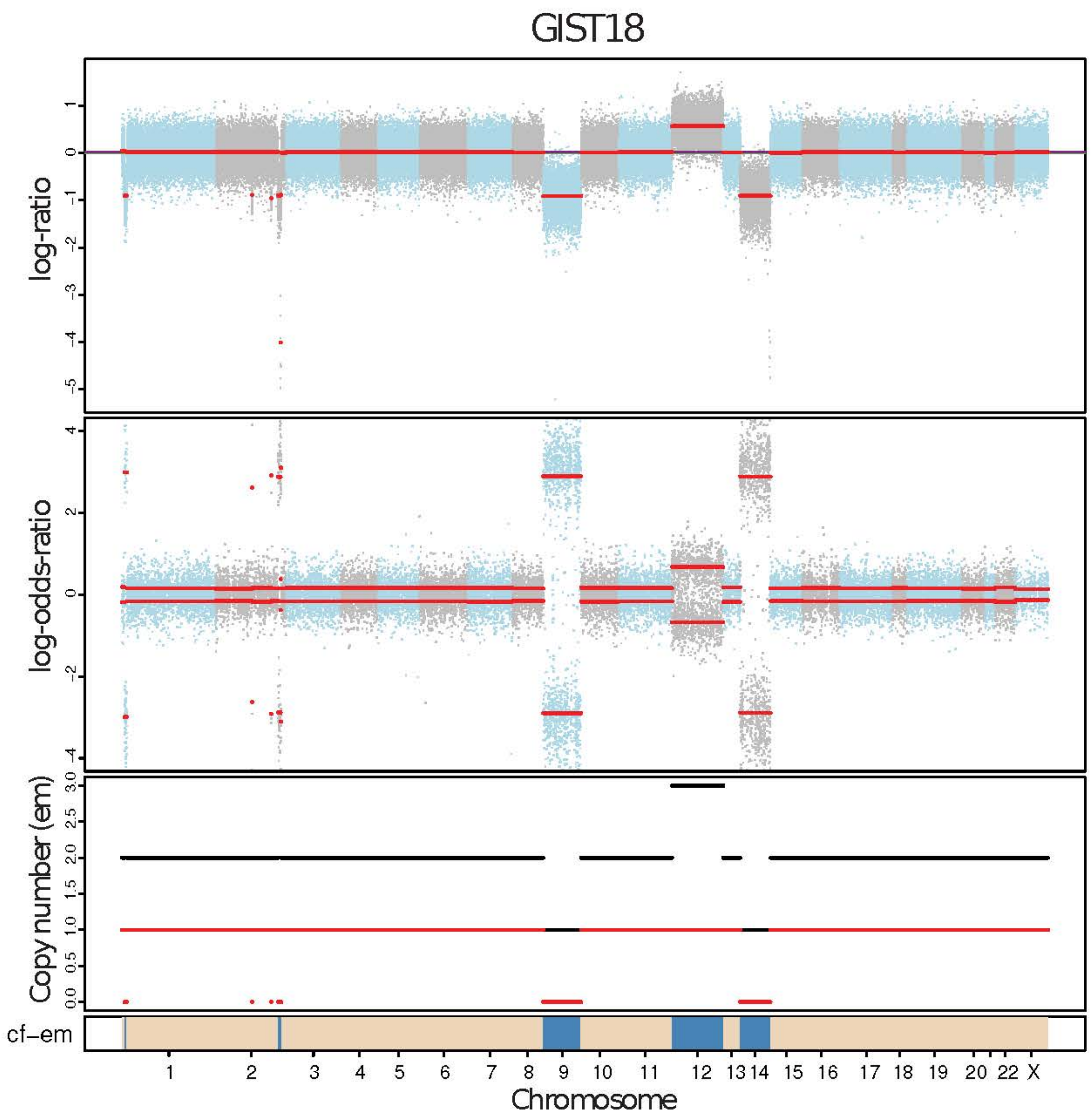

[Purity: 0.94, Ploidy: 1.97, Diploid LogR: 0.02, Log likelihood: 49.44]

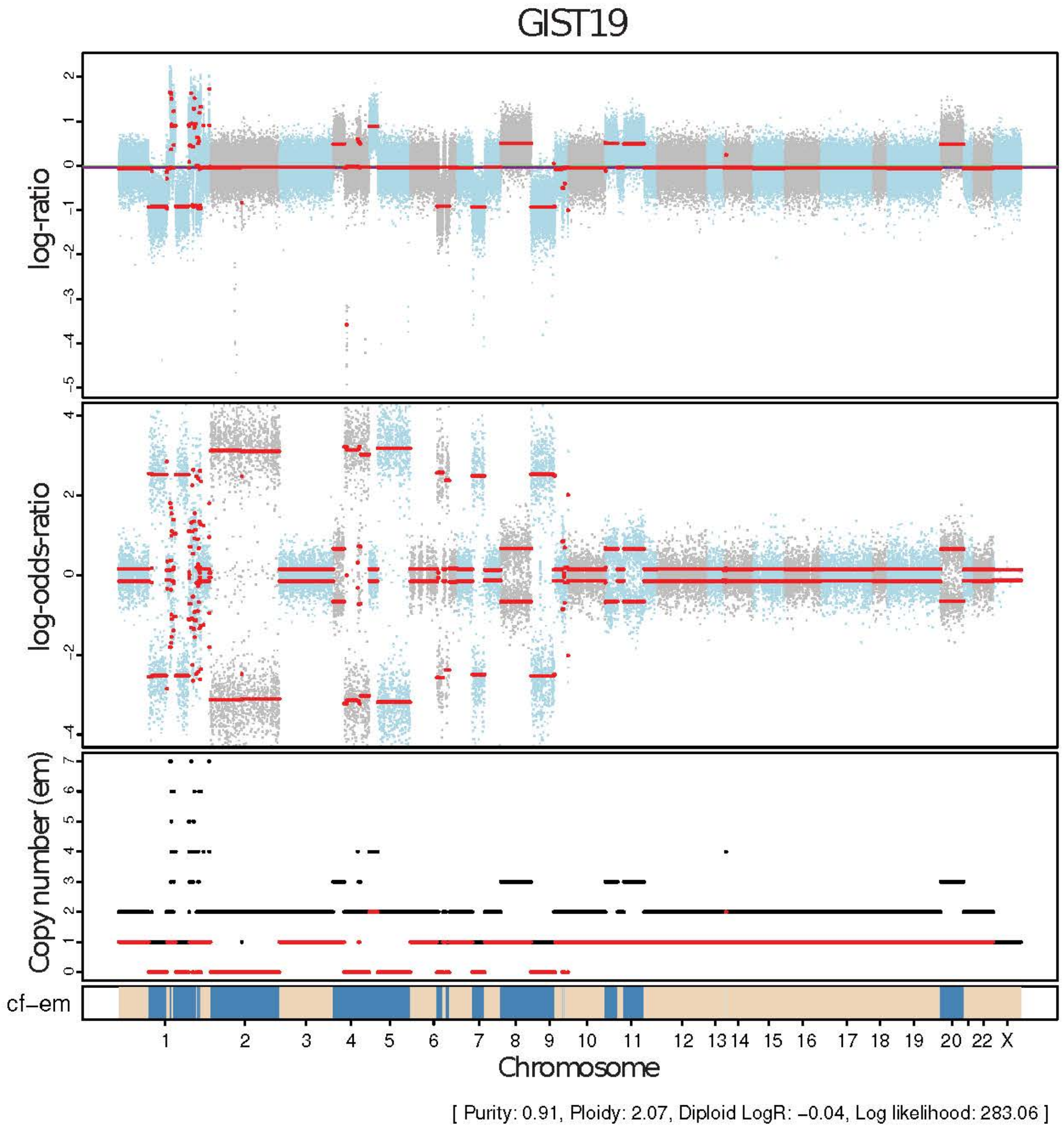
GIST19
log-ratio
log-odds-ratio
Copy number (em)
cf-em
1 2 3 4 5 6 7 8 9 10 11 12 13 14 15 16 17 18 19 20 22 X
Chromosome
[Purity: 0.91, Ploidy: 2.07, Diploid LogR: -0.04, Log likelihood: 283.06]

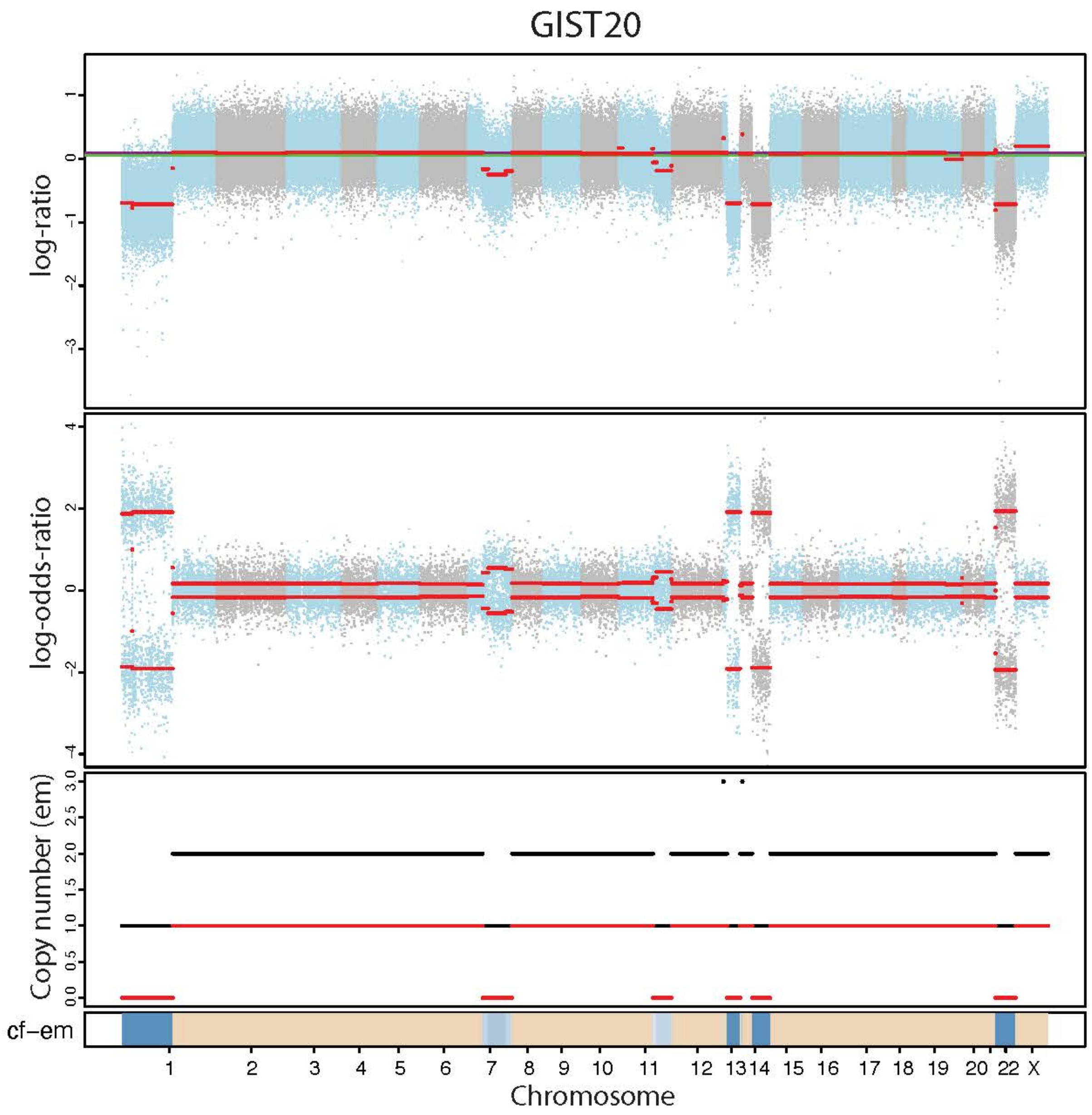

[Purity: 0.85, Ploidy: 1.85, Diploid LogR: 0.09, Log likelihood: 135.35]

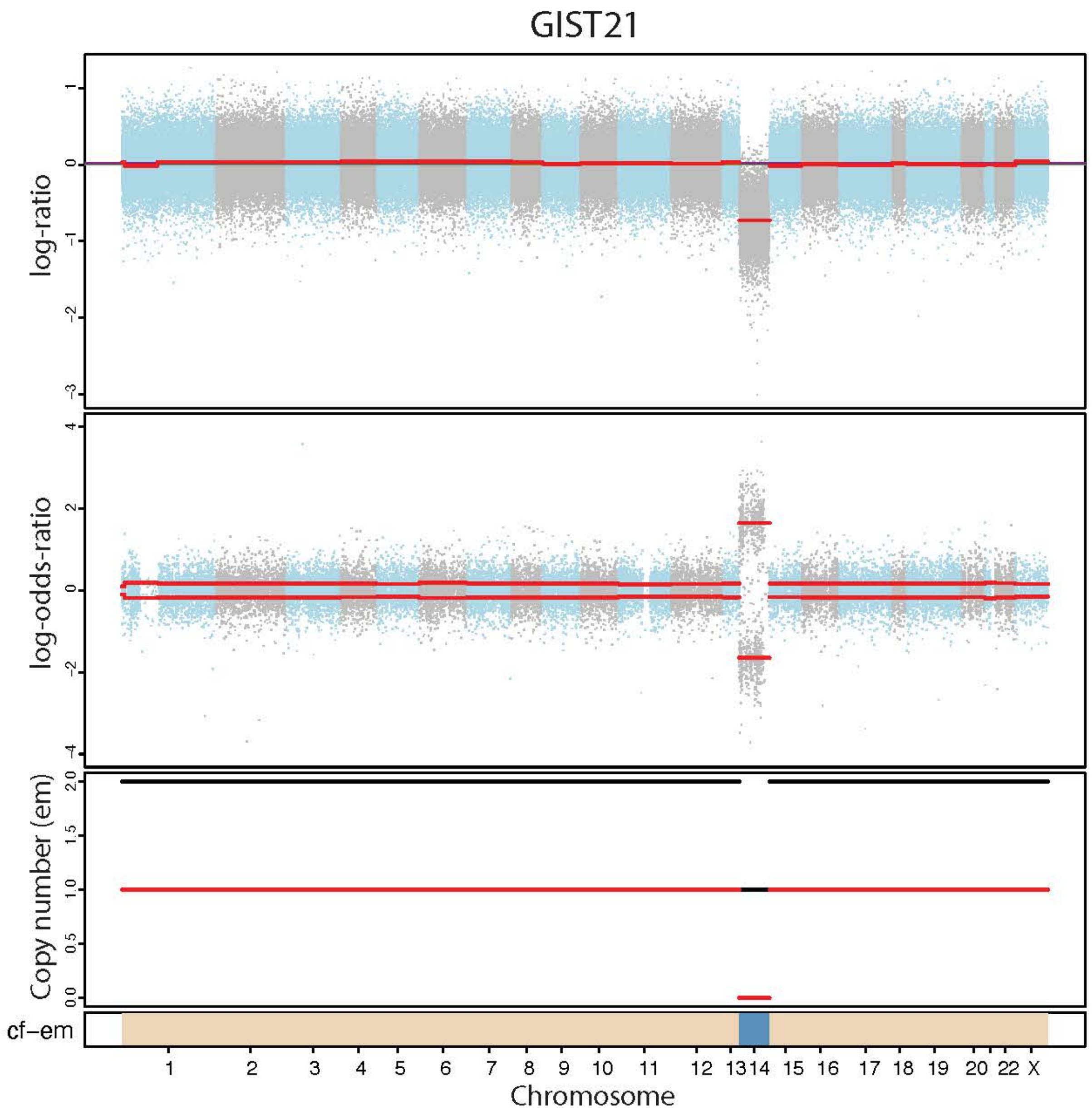

[Purity: 0.81, Ploidy: 1.96, Diploid LogR: 0.02, Log likelihood: 26.71]

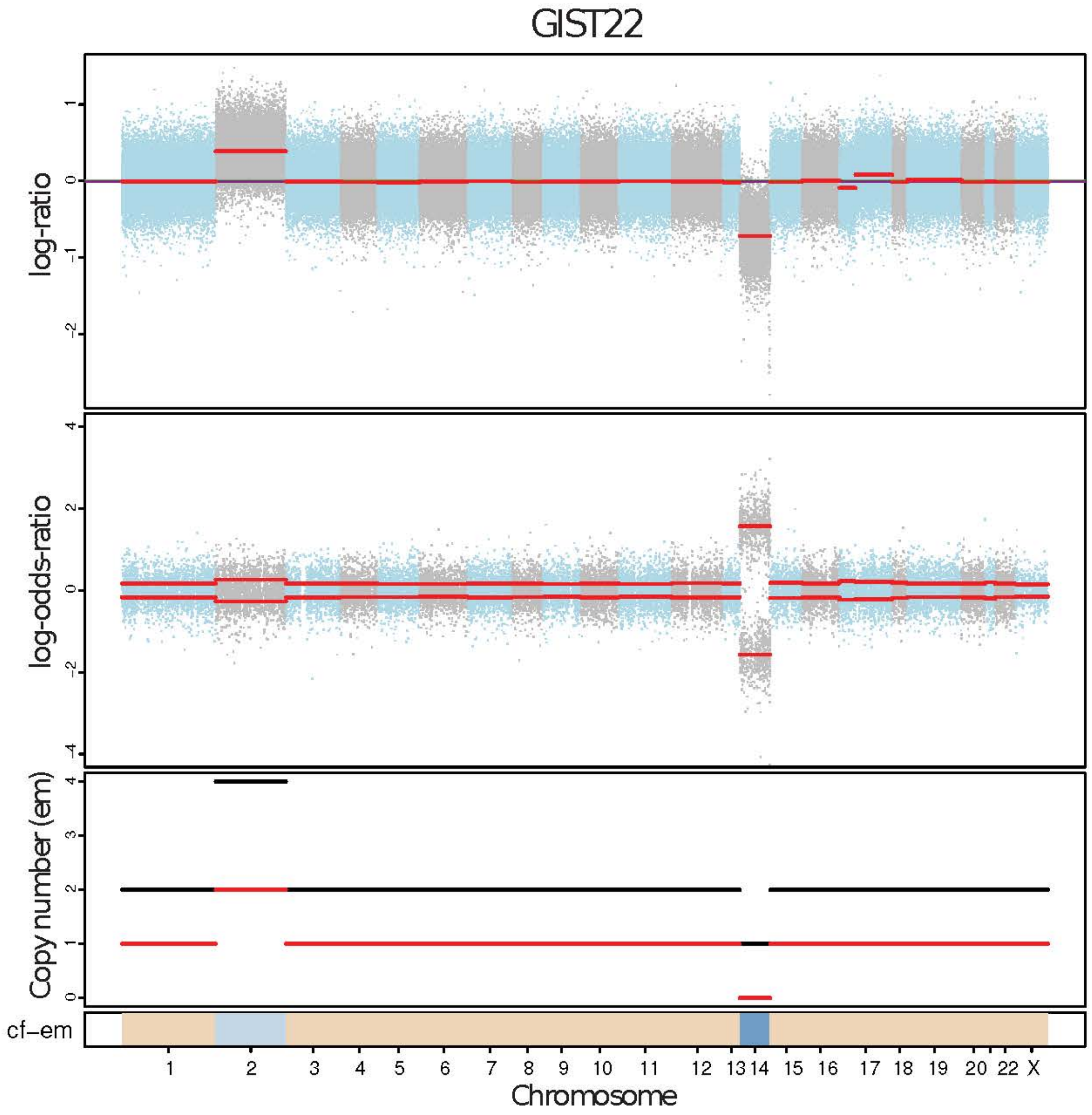

[Purity: 0.71, Ploidy: 2.02, Diploid LogR: −0.01, Log likelihood: 67.16]

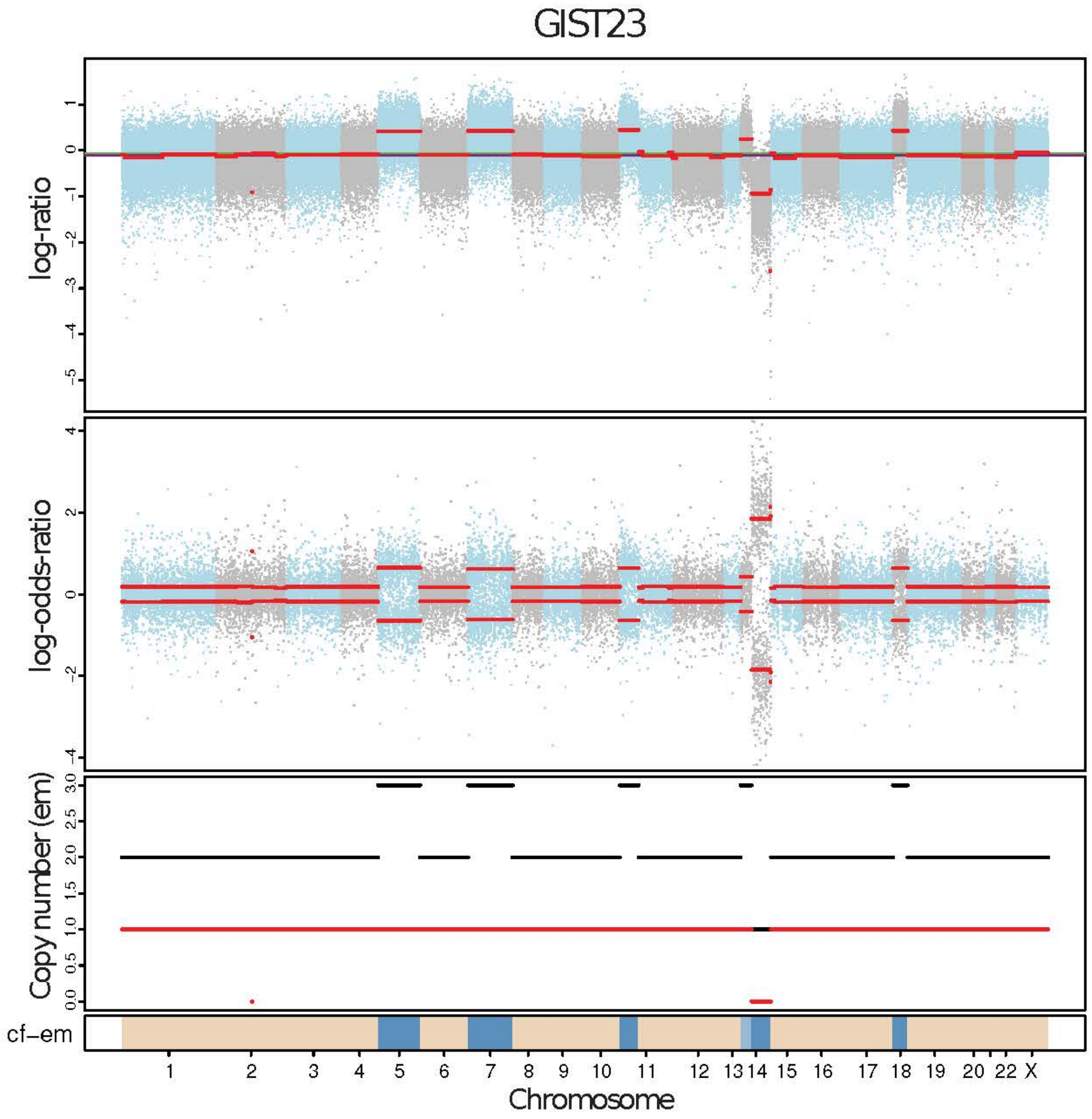

[Purity: 0.51, Ploidy: 1.75, Diploid LogR: 0.1, Log likelihood: 80.18]

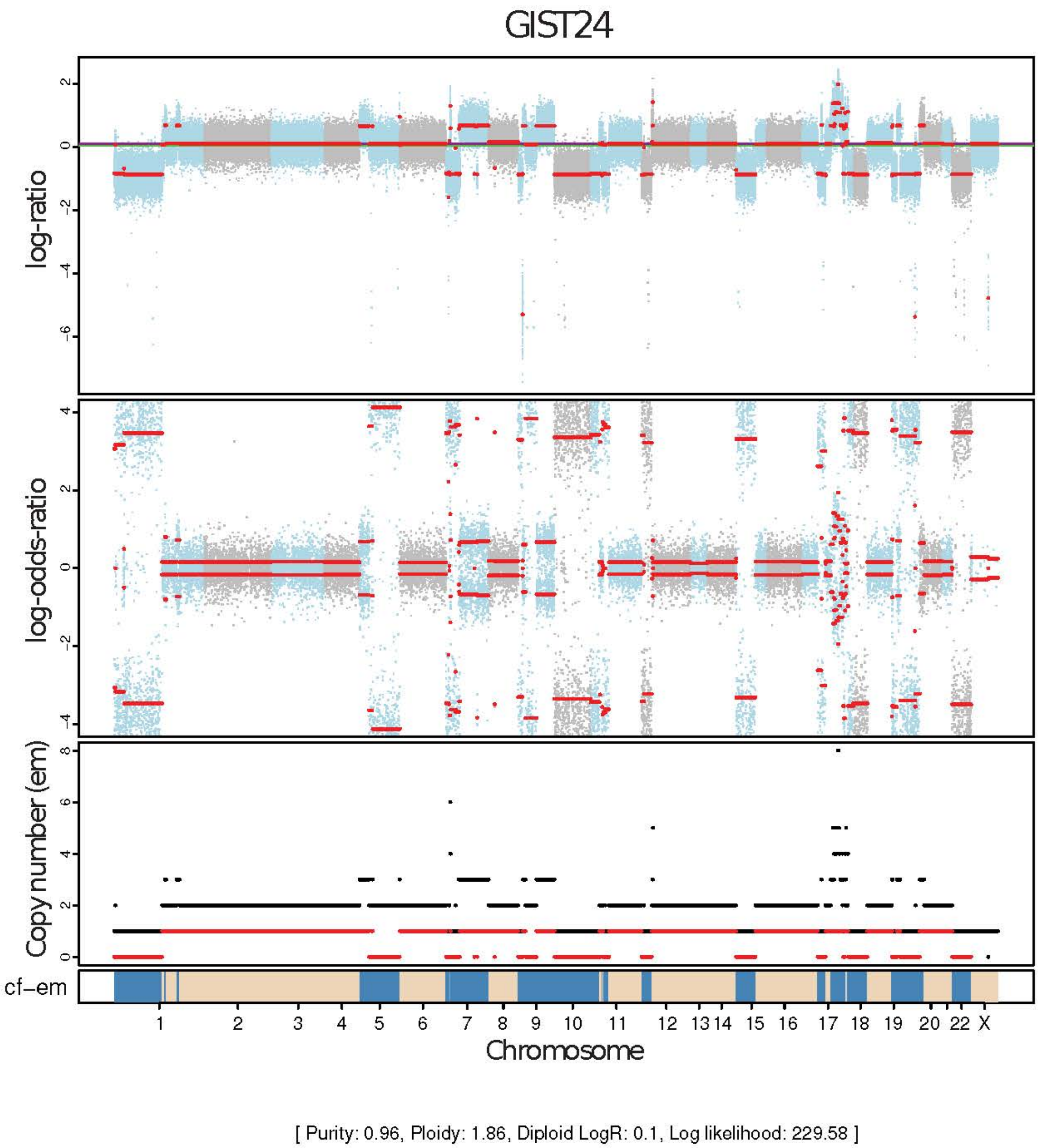

Supplementary File S5. Plots showing copy-number, LOH and ploidy for each of the GIST samples generated using FACETS.
