# Supplementary material for: Chromosomal instability and a deregulated cell cycle are intrinsic features of high‐risk gastrointestinal stromal tumours with a metastatic potential
Source: Mol Oncol. 2023 Sep 3;17(11):2432–50. doi: 10.1002/1878-0261.13514 (PMC10620130; doi:10.1002/1878-0261.13514)
Supplement: Supplementary file 7 — Data S7. Hierarchical clustering of GIST based on mRNA expression. [file MOL2-17-2432-s006.pdf]

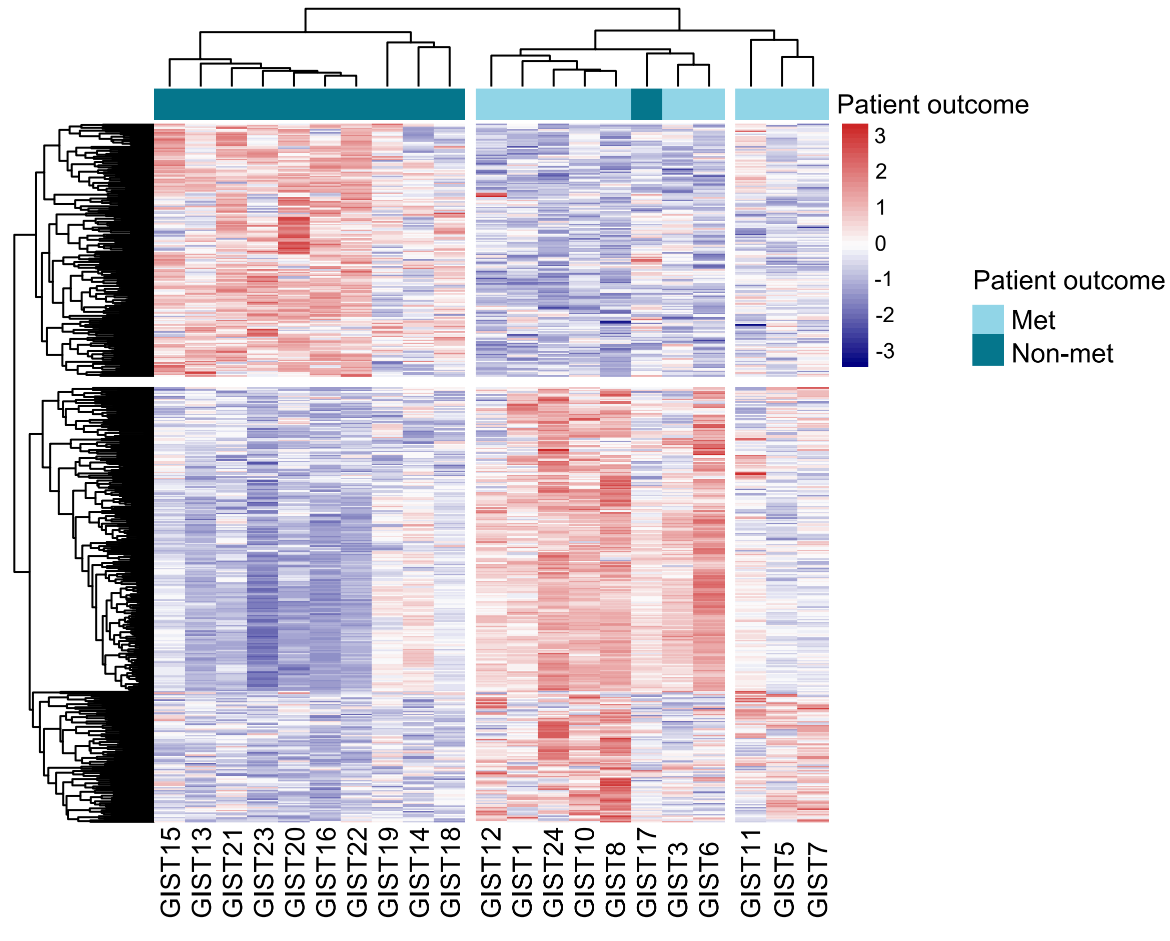

Supplementary File S7. Hierarchical clustering of GIST based on mRNA expression. Unsupervised clustering based on normalized expression data of 487 differentially expressed genes. Cut-off p-value<0.05 and |logFC|>1. Pearson Correlation distance and complete linkage with Z-score transformation.
