# Supplementary material for: Chromosomal instability and a deregulated cell cycle are intrinsic features of high‐risk gastrointestinal stromal tumours with a metastatic potential
Source: Mol Oncol. 2023 Sep 3;17(11):2432–50. doi: 10.1002/1878-0261.13514 (PMC10620130; doi:10.1002/1878-0261.13514)
Supplement: Supplementary file 11 — Data S11. Hierarchical clustering analysis of miRNA expression. [file MOL2-17-2432-s008.pdf]

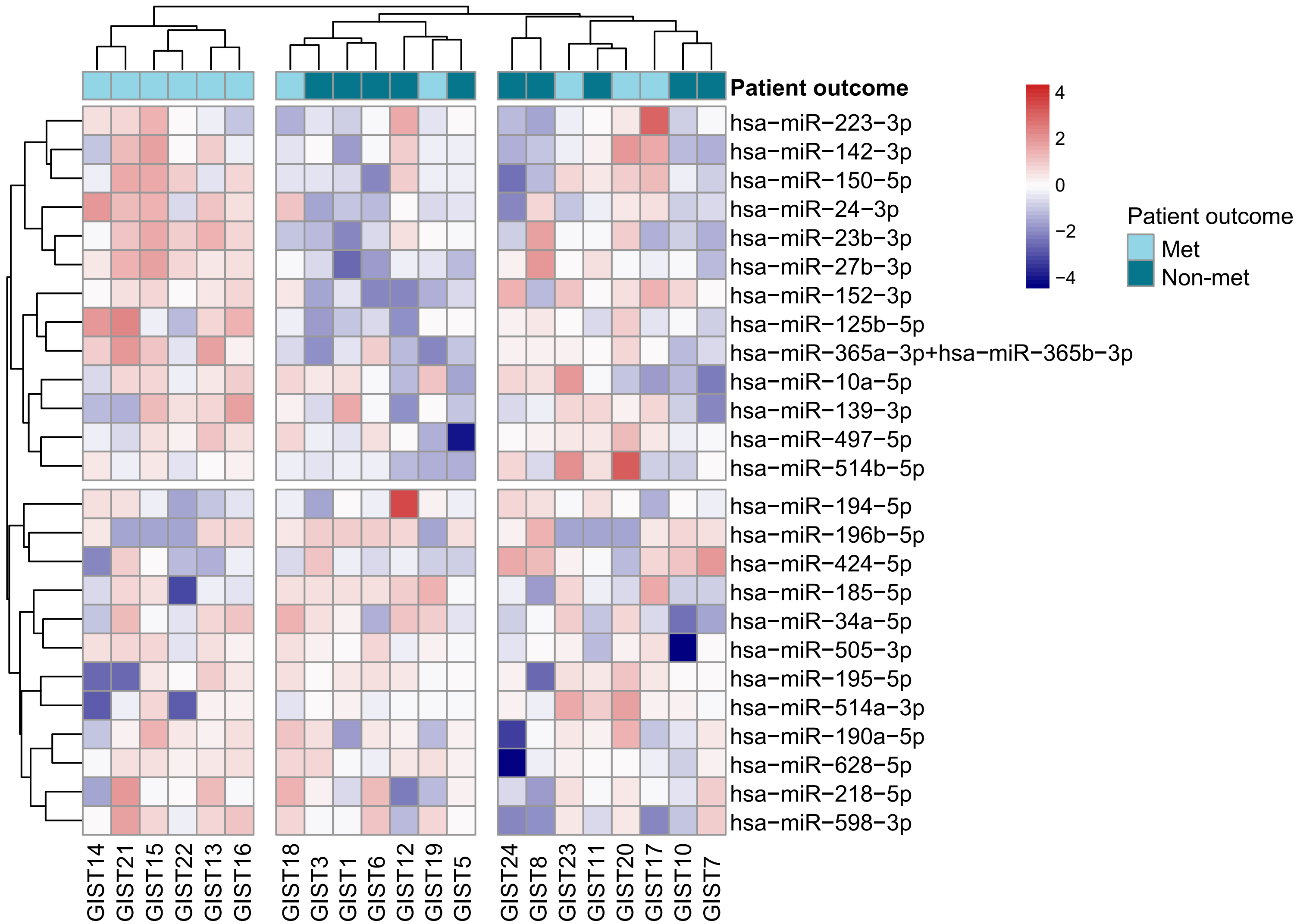

Supplementary File S11. Hierarchical clustering analysis of miRNA expression. Unsupervised clustering based on normalized expression data of 25 differentially expressed miRNAs. Cut-off p-value<0.05 and |logFC|>1, median expression>45 in at least one high-risk group. Pearson Correlation distance and complete linkage with Z-score transformation.
